# The Physical Chemistry and Chemical Physics (PCCP) Section of the *International Journal of Molecular Sciences* in Its Publications: The First 300 Thematic Articles in the First 3 Years

**DOI:** 10.3390/ijms23010241

**Published:** 2021-12-27

**Authors:** Oleg V. Mikhailov

**Affiliations:** Department of Analytical Chemistry, Certification and Quality Management, Kazan National Research Technological University, K. Marx Street 68, 420015 Kazan, Russia; olegmkhlv@gmail.com

## 1. Introduction

The Physical Chemistry and Chemical Physics Section (PCCP Section) is one of the youngest among the sections of the *International Journal of Molecular Sciences* (*IJMS*)—the year 2021 will only mark three years since its inception. Despite its very small “age”, it has already gained wide popularity among specialists in the above-mentioned branch of science, which is at the intersection of Chemistry and Physics. During the entire period of its existence, over 300 articles have been published within this section, the first of which was dated 25 October 2018, the last 14 May 2021 (at the time of this writing), and the growth rates of their number have a distinct tendency to increase over time. To a large extent, this increase is facilitated by several factors, among which, first of all, is worth noting the indexation of the *International Journal of Molecular Sciences* in both of the most authoritative international citation databases—Web of Science and Scopus (which have very high values of impact factors that, in general, also increase from year to year). The journal has a fast peer-review process of submitted articles (within no more than one month) and a very fast—even by modern standards!—publication of articles after the official decision of acceptance for publication (within one week). It is also important that all articles, except for the articles that were published at different times in *IJMS* (as well as in the PCCP Section), have open access on the internet, thanks to which they can be accessed by a very wide range of users (and not interested researchers only). Kind words also deserve the design of articles published in the journal, which has no analogues in other journals (with the exception of the journals only published by MDPI, the design of which is sustained in the same original style).

The statistical data on the 300 articles considered in this review are presented in Table 1. As it can be seen, the vast majority of them (over 80%) are original articles; review articles are presented in much smaller numbers. It is noteworthy that in the list of publications, there are practically no short messages and editorial articles. The last circumstance is not very clear. First, as mentioned above, the journals of the MDPI Publishing House, in general, and the *International Journal of Molecular Science*, in particular, are distinguished by a very high publication speed, which is very important for articles of Communications type. Secondly, in the MDPI journals, the organization of various thematic Special Issues has recently become widespread (of which, during the time period considered in this review, from October 2018 to May 2021, about fifty were organized, and about a third of which have already been completed) and, it would seem, the guest editors of these issues should have submitted a fairly large number of Editorials. Despite this, so far, only five such articles have been published, and two of them were written by the author of this review.

Currently, within the PCCP section, nine Thematic Areas have been officially identified, to which, in principle, articles under its auspices should relate, namely (see the Physical Chemistry & Chemical Physics Section of the *IJMS* website https://www.mdpi.com/journal/ijms/sections/PCCP (accessed on 5 December 2021).

1. Intermolecular forces that act upon the physical properties of materials;
2. Reaction kinetics on the rate of a reaction;
3. The identity of ions and the electrical conductivity of materials;
4. Surface science and the electrochemistry of cell membranes;
5. Probing the structure and dynamics of ions, free radicals, polymers, clusters, and molecules;
6. Chemical structures and reactions at the quantum mechanical level;
7. The structure and reactivity of gas-phase ions and radicals;
8. Energy/charge transfer dynamics in organic/inorganic materials;
9. Physical processes in nanomaterials.

In principle, it is possible to systematize the articles published within the section using one of two options—either chronological, which is based on the time of publication of the article in *IJMS*, or thematic, whereby all articles are sorted into separate groups under their assignment to or from the scientific directions listed above. In the given review, the thematic principle of sorting the material will be used, and at least a small, but separate, paragraph will be devoted to each of the above nine positions. Additionally, quite numerous Special Issues of *IJMS* deserve separate consideration, the part of which has already been completed (see https://www.mdpi.com/journal/ijms/special_issues?section_id=447&search=&sort=deadline&view=closed&page_count=100 (accessed on 5 December 2021), and the others are ongoing and are scheduled for completion in 2021 (see https://www.mdpi.com/journal/ijms/special_issues?page_count=10&search=&section_id=447&sort=deadline&view=open&page_no=3 (accessed on 5 December 2021). In general, this review will be devoted to the first 300 articles that were published in the *International Journal of Molecular Sciences*, in the above period. A list of these 300 articles in chronological order is presented in the Supplementary Materials to this article. Moreover, at least some of these articles can be attributed not to one, but two or even more of these nine positions.

## 2. Articles on the Various Directions

Since the journal title itself, as well as the name of the PCCP Section, covers a very wide area of physicochemical processes and related objects of study, it is quite natural to expect that the topics of the articles published in it will differ in a rather significant variety, comparable with what takes place in specialized journals in the field of Chemical Science (for example, *Physical Chemistry* (ACS), *PhysChemChemPhys* (Wiley), *Chemical Physics Letters* (Elsevier), and *Perkin Transactions* (RSC)). The reality, however, surpassed all these expectations, since, in general, the topics of these first 300 articles turned out to be so diverse, that it is very difficult (if not impossible) to combine them into any coherent story. In this connection, regarding both the content of individual articles and the content of those thematic sections to which they were referred by us, we will generally have to limit ourselves to only brief comments.

### 2.1. Intermolecular Forces That Act upon the Physical Properties of Materials

Articles [1,2,3,4,5,6,7,8,9,10,11,12,13,14,15,16,17,18,19,20,21,22,23,24,25] can be attributed to the first of the thematic areas indicated in the PCCP Section, and the distribution of these articles into the above categories is presented in Table 2. As it can be seen, of the four types of articles established by the magazine’s standards (**Reviews**, Originals, *Communications*, Editorials), only the first two are presented here. The first of them [1], dated early November 2018, was devoted to the study of the intermolecular interactions that contribute to the stability of the tear film; it was the first to study the conformation of the hydrocarbon chain and rheology of meibum from babies. Articles [2,3,6] can also be referred to the biology-medical direction in the Thematic Area 1; the first two of them are devoted to the problem of the so-called molecular recognition of organic compounds, and the third to the specific interaction of peptide molecules in aqueous solutions. The most significant place in this thematic area belongs to works in which mesoporous micro- and nano-spherical particles, and the intermolecular forces associated with them, appear as the object of research [12,15,16,17,20]. These particles are also reviewed in [21], which provides the latest information on mesoporous silica nanoparticles (MSN), functionalized to target subcellular compartments, with an emphasis on the cytoplasm, mitochondria, and nucleus, which are now widely used as drug carriers due to their unique physical–chemical characteristics. To some extent, this also includes the other two review articles from this thematic area, namely [9,22], the first of which addresses the problem of the existence of the so-called exclusion zones (EZ), namely, a layer of water in which plastic microspheres are repelled from hydrophilic surfaces, the second of which explores the systematization of processes known in the literature for obtaining microparticles of insoluble β-glucans from spent brewer’s yeast and gives an idea of how a by-product of brewing can be converted into potential food items. To the specifics of intermolecular interactions in systems containing surfactants, publications [4,12,18] were devoted; intermolecular interactions, in which the formation of hydrogen bonds plays a key role, are discussed to varying degrees in [5,23,25]. Two publications [7,19] provide data on the specific interactions in “guest-host” systems. Occasionally, there are works devoted to other aspects related to intermolecular interactions, in particular, to the formation of liquid crystal systems [8], supercooled bulk water [11], and liquid-liquid extraction aimed at separating individual heavy metals from aqueous solutions [10].

### 2.2. Reaction Kinetics on the Rate of a Reaction

Thematic Area 2 includes 22 articles, namely [26,27,28,29,30,31,32,33,34,35,36,37,38,39,40,41,42,43,44,45,46,47], the typification of which is shown in Table 3. With some degree of conventionality, it is possible to subdivide the articles published here into two main groups, namely, articles devoted to the study of the kinetics of processes in which any chemical transformations are decisive, and articles devoted to the study of the kinetics of processes in which chemical transformations are either not decisive or absent altogether. Among the articles of the first group, in turn, two categories can be distinguished, in the first of which are the clear majority; the processes related to them were considered in the articles [29,30,31,32,33,35,37,42,43,44,45,46,47]. For example, in [29], the kinetics of the aldol condensation of benzaldehyde and heptanal in liquid-phase systems using various solvents was characterized, and a very pronounced effect of the solvent was noted for a given set of identical kinetic parameters (i.e., the same kinetics of the liquid phase can lead to different values of conversion and yield of reaction products depending on the choice of solvent). It was shown that the subsequent assessment of the parameters without taking into account the activity coefficients led to a pronounced deviation from the “true” kinetics up to 30 times. In [31], a kinetic study of the solvolysis of *o*- and *p*-isomeric nitrobenzyl bromides and *o*-nitrobenzyl chloride was carried out in a wide range of solvents at different temperatures. An interesting study was carried out in the article [37], in which the oxidation of anthracene by hydroxyl radicals obtained by the reaction of 5,10,15,20-tetrakis(4-carboxyphenyl) porphyrin of Fe(III) with hydrogen peroxide under visible radiation in a nitrogen atmosphere was considered. From the list of articles cited above, in our opinion, one should also mention [30,43,45], which are remarkable in that, in each of them, the kinetics of any of the biochemical processes was studied (for example, in [45], the dissolution of self-organizing model peptide fibrils after quenching by dilution using the isothermal titration calorimetry method). Only works [34,39,40] are devoted to the study of the kinetics of reactions with the participation of inorganic compounds. In all these articles, however, some chemical reactions took place in one way or another, i.e., they all belong to the first of the above two groups of articles. The articles of the second group are presented in only [36,38] and are devoted to sorption processes: in [36], various experimental conditions were tested to optimize the removal of Hg(II) by the bark of *Eucalyptus globulus* and, in [38], titanium(IV) hydrogen phosphate was used as a sorbent. Among the works of a purely theoretical plan, one can note [41], in which the authors investigated how the size, the number, and the spatial arrangement of identical nonoverlapping reactive patches on a sphere influenced the overall reaction kinetics of the bimolecular diffusion-limited (or diffusion-controlled) reactions that occurred between the patches and the reactants diffusing around the sphere. It should be noted, at the end of this paragraph, that in the Thematic Area 2 only one of the Original Articles is presented, while there are no other three types of articles; this is the only paragraph with such a typification.

### 2.3. The Identity of Ions and the Electrical Conductivity of Materials

The third of the directions officially accepted in the PCCP Section, is represented by only 11 articles [48,49,50,51,52,53,54,55,56,57,58], including 9 Originals and 2 Reviews. Their distribution by years of publication is presented in Table 4. It is worth noting that the content of this paragraph is actually connected only with the second of the directions indicated in the title, namely with electrical conductivity and/or electrochemical processes in which this parameter is measured in one way or another. In our opinion, the most significant in this direction are two review articles [54,55] published in 2020 and devoted to ionic liquids. The first of them [54], describes a variety of structures, properties, and examples of the use of chiral ionic liquids as new chiral solid materials and chiral components of an anisotropic medium, providing chiral recognition of enantiomeric analytes, which is useful in, for instance, liquid chromatography and countercurrent chromatography. Unlike [54], review article [55] contains a critical review of modern literature on the structure–property relationship of a very wide range of such liquid-phase systems; in particular, it revises modern “structure–property” correlations for a wide range of the above objects, within which the relationship between the so-called transport properties of these liquids (viscosity, diffusion, and electrical conductivity) and free volume are analyzed and discussed at the molecular level. It also presents new perspectives based on existing data. In addition to these review articles, research articles [56,57,58] are devoted to ionic liquids. The rest of the publications of this section are completely dissimilar from each other in thematic respect, and it is very difficult (if not possible at all) to find something in common between them (except that they can all be attributed to the field of electrochemistry). Nevertheless, it is worth noting an interesting work [48], in which, to optimize the physicochemical properties of phthalocyanine (PC), its behavior in particles of the triple helix of glucan curdlan (CUR), in which PC is retained inside CUR molecules, was studied. The electrical conductivity of the CUR–PC solution was found to be higher than the conductivity of the CUR solution or PC dispersion, indicating that CUR–PC is more soluble in water than PC; in addition, CUR–PC has proven to be very stable in aquatic environments.

### 2.4. Surface Science and the Electrochemistry of Cell Membranes

This thematic area is associated with articles [12,15,20,21,26,50,59,60,61,62,63,64,65,66,67,68,69,70,71,72,73,74,75,76,77,78,79,80,81,82,83,84,85,86,87,88,89,90,91,92,93,94,95,96,97,98,99,100,101], of which there are a total of 50. In contrast to the areas already discussed above, articles of all four types are presented here (Table 5), although, here, too, the Original constitutes a clear majority over the other 3 types of articles. The dominant feature of this thematic area was articles devoted to cell membranes, in general, and their electrochemistry, in particular; articles [26,50,59,60,61,62,63,64,65,66,67,68,69,70,71,72,73,74,75,76,77,78,79,80,81,82,84,85,86,87,88,89,90,92,93,95,99,100] are devoted to this specific topic. In this connection, it is worth noting that this “membrane topic” turned out to be, generally, the most popular in comparison with all the others presented in the PCCP Section of the *IJMS*, within the array of 300 articles we are considering. The range of issues discussed in these publications is very wide: from integrated mechanical and fluid dynamics simulations to investigate the trans-membrane pressure effects on deformation, flow and mass transfer for a profiled membrane–fluid channel system with geometrical and mechanical features and fluid velocities representative of electrodialysis/reverse electrodialysis conditions [62] to the modification of the surface of polypropylene membranes with radiofrequency plasma [66]; from the synthesis of a new hydroxide exchange membrane for zinc-air batteries [50] to the assessment of the salt permeability of three industrial cation-exchange membranes of various morphologies in aqueous NaCl solutions [87]. However, electrochemical aspects to one degree or another were considered only in works [26,50,60,67,69,70,75,78,79,95]. The most significant publications here are reviews related to membrane technologies [59,73,84,86]. The first of them [59] is devoted to the so-called membranes with designed topography (profiled, corrugated, patterned, and notched membranes) that are gaining increasing academic and industrial attention and recognition as a quite viable alternative to flat membranes. In [73], the problems of evaluating methodologies and experimental methods aimed at solving such an important matter is the distribution and concentration of the so-called transmembrane proteins that make up the membrane of the nucleus of eukaryotic cells. The subject of work [84] is related to ion-exchange membranes, which find many applications in water desalination, electrolysis, chemical, and biological technologies, solving problems related to human health, energy, environment, and other fields; it discusses the problems associated with the development of these membranes and the prospects for further development in this sphere of research. Standing apart, against this background, is the review [86], which provides a comprehensive analysis of the relationship between aquaporins (water-specific membrane channel proteins that regulate water homeostasis in cells and the body) and rhinological conditions; it focuses on the analysis of the mechanisms that connect them with each other. Since all of the above articles were included in two special issues, “Ion and Molecule Transport in Membrane Systems” and “Ion and Molecule Transport in Membrane Systems 2.0”, it is quite understandable that there are two editorials among them, namely [99,100]. Only less than 10 articles belong to the surface sciences proper, namely [12,15,20,21,83,91,96,97,98], and the topics and objects of research are also very diverse. For example, in [12], the synthesis of Mobil Composition of Matter 41 (MCM-41) mesoporous silica nanoparticles of controlled sizes and porous structure has been performed. Three articles were related to the study of mesoporous silica nanoparticles; so, in the first of them [20], it was shown how, after coating mesoporous silica particles with various polyelectrolytes or proteins, it is possible to produce completely individualized hollow shells for encapsulating various drugs; in the second [96], mesoporous silica with large pores were developed for the transfer of large biomolecules and their release from these pores by changing the acidity of the medium; and, in the third [97], two different types of ordered mesoporous nanoparticles, using the passive diffusion method. It should be noted that among these publications, there is also a specialized review [21], which is also devoted to such mesoporous silica nanoparticles, which are functionalized to target them to subcellular compartments, with an emphasis on the cytoplasm, mitochondria, and the nucleus.

### 2.5. Probing the Structure and Dynamics of Ions, Free Radicals, Polymers, Clusters, and Molecules

This area is the largest in terms of the number of related publications and includes 91 articles, namely [1,2,3,102,103,104,105,106,107,108,109,110,111,112,113,114,115,116,117,118,119,120,121,122,123,124,125,126,127,128,129,130,131,132,133,134,135,136,137,138,139,140,141,142,143,144,145,146,147,148,149,150,151,152,153,154,155,156,157,158,159,160,161,162,163,164,165,166,167,168,169,170,171,172,173,174,175,176,177,178,179,180,181,182,183,184,185,186,187,188,189]. Such a predominance of this direction against the background of the others is quite natural, since its subject covers the widest range of objects under study in comparison with any other. The distribution of related articles according to the above types is presented in Table 6. As can be seen, there are three types of articles out of four. In principle, it is possible that the systematization of these articles can be carried out with an orientation towards two main classes of chemical compounds (organic and inorganic), with an orientation towards the objects indicated in the name Thematic Area 5 (i.e., ions, free radicals, polymers, clusters, and molecules), or with a combination of both of these options. The last one seems to be the most detailed, and it is precisely this that we will try to implement in the course of our narrative. As it should be expected, a priori, most of the publications in this direction are devoted to organic compounds, and primarily organic molecules; these include, with varying degrees of correctness, publications [1,2,3,102,103,104,106,108,112,117,118,119,121,124,127,128,130,131,132,133,135,138,139,140,141,142,143,144,146,147,148,151,153,154,156,157,164,165,166,168,171,172,174,176,178,180,183,185,186]. Much less space is occupied by the works related to polymeric (in fact, to a high molecular weight) compounds [113,114,134,149,152,160,173,177,179,181,182]; moreover, they are all, in one way or another, related to the study of proteins (polypeptides). There are practically no works on the structure/dynamics of synthetic polymers in the list of these 300 publications; the only exception, and even then with a stretch, that can be considered is article [177], in which polyurethane fibers are one of the objects of research. In fact, in this list, there are no articles in which the object of study would be ions, free radicals, and clusters, although, with a certain stretch, they can be attributed to them [118,155,169], in particular, publication [118] devoted to the N,N–di(4–Bromo)nitrenium. Be that as it may, the objects in these articles are distinguished by an exceptional variety, and it is extremely difficult, if not impossible, to carry out a more detailed systematization of them (and even more so, to give details of their content in this review article). Nevertheless, in our opinion, it is worth noting here the publications devoted to Schiff’s bases, which are presented both by original research articles [112,121,130,137,143,147,156,163] and by two reviews; the first of which [125] presents actinide (5f-elements) complexes, and the second [142], the complexes of platinum metals. Moreover, noteworthy, are the articles, the problems of which are related to another important class of organic compounds, both theoretically and applied, namely, with various porphyrins and their derivatives [135,140,145,168,169,175,178]. In this regard, a review [141] devoted to porphyrin nanoparticles for the phototherapeutic treatment of oncological diseases is of considerable interest. (Since we are talking about such an important problem of modern human civilization as the treatment and prevention of various diseases, it is worth mentioning article [151], in which polymorphic forms of valinomycin were obtained and characterized by various methods, which seem to be promising for the treatment of COVID-19). In addition to the three reviews named above, others are presented in this thematic area. For example, in review [139], the subject of discussion was photoacoustic visualization using compounds that can be considered as peculiar precursors of porphyrins, namely tetrapyrroles, and in review [106], macrocyclic compounds that are important in modern supramolecular chemistry, namely cyclodextrins, cucurbiturils, calixarenes, and cillarenes. Review [158] presents information that is very rarely found in the modern chemical literature, namely, about azulene derivatives with heterocyclic fragments in the molecule, the specifics of their synthesis, and the prospects for their application in materials science. In review [161], devoted to the so-called macrolide antibiotics, a description of advanced physicochemical methods for elucidating the structure and interaction of macrolide antibiotics in solid state and solution is presented, and their main advantages and disadvantages are considered. Finally, review [176] systematizes and characterizes the latest advances in precision rotational-vibrational spectroscopy of neutral (mainly organic) molecules, within which spectra are obtained by using infrared frequency combs, either as direct probe sources or as ultra-accurate optical rulers.

As for the publications devoted to the structure and dynamics of inorganic compounds, for most of them the objects of study were substances that, in principle, can be classified as clusters in the broad sense of the word; these are [105,109,115,116,120,122,123,124,126,159]. In five of these ten articles [115,116,120,124,159], the objects of study were elemental gold nanoparticles (which, in principle, can be considered as large clusters, in the narrow sense of the word, due to the presence of metal–metal chemical bonds in their structural units), which were either in a free state [115,124,159] or were covered with a layer of some chemical compound (i.e., they were the core of the “core-shell” system [116,120]). In all these works, gold nanoparticles were used to solve any problems in medicine and/or pharmacology. In [105], the process of the “clustering” of water molecules was considered and a new mechanism for the formation and destruction of giant water clusters with sizes from ten to hundreds of micrometers was proposed. Neutral, mono-, and di-anionic zirconium-doped silicon clusters having a ZrSi_n_^0/–/2−^ composition, where n varied from 6 to 16, were investigated in the article [109]. Other objects from the list mentioned in the title of this thematic area were studied, only sporadically. The structure and dynamics of the molecules of inorganic compounds were considered in publications [107,111,170,175], inorganic ions in [145,167,184], and inorganic polymers only in article [110]. In [107], this object was methane hydrate; in [111], a Cu(II) complex with polyvinyl alcohol; in [170], a heteronuclear complex of Cu(II) and Cd(II) with a “cubane” core; and in [175], Co(III) with ammonia or 1,10-phenanthroline. The articles [118,184] should be noted among the works devoted to inorganic ions. In the first of them, as it was already mentioned earlier, the structure of the *N*,*N*–di(4–bromo) nitrenium ion was considered, and, in the second, the processes of chemisorption of s-element ions of group I in the periodic table with thiacalix[4]arene mono crown. Article [110], indicated above is devoted to the processes of oligomerization of silicic acids in aqueous media. The only review [126] related to the structure and dynamics of inorganic compounds is devoted to a discussion of the processes of enhancing the efficiency of reactive oxygen species associated with the participation of elemental metal nanoparticles, as well as the mechanisms of these processes and factors influencing these processes.

In conclusion of Thematic Area 5, it is worth mentioning review articles [187,188,189], which stand apart from other articles, since they are no longer devoted to specific organic or inorganic compounds, but general problems related to the molecular modeling of the structures of chemical compounds. In particular, Ref. [187] discusses the differences, relationships, and potential interactions between the three algorithmic directions to create more efficient and reliable algorithms for such simulations in complex molecular systems.

### 2.6. Chemical Structures and Reactions at the Quantum Mechanical Level

This direction is represented by 39 articles [28,35,129,134,136,190,191,192,193,194,195,196,197,198,199,200,201,202,203,204,205,206,207,208,209,210,211,212,213,214,215,216,217,218,219,220,221,222,223]. Their distribution, according to the above types, is presented in Table 7. In principle, they can be divided into three groups; namely, (*a*) articles that deal with purely theoretical problems associated with assessing the quality of existing quantum–chemical calculation methods, their application for calculating certain other characteristics of the molecular structures and reactions of their formation; (*b*) articles devoted to the calculation of the parameters of molecular structures and the kinetics of organic compounds; and (*c*) articles devoted to the calculation of the parameters of molecular structures and the kinetics of inorganic and coordination compounds. In all these articles, studies (or their systematization in the case of review articles [194,220,222,223]) were carried out using either the most popular method nowadays (the DFT method), with functionals of different levels (for instance, B3LYP, OPBE, and M06), or the even more complex ab initio CCSD method. The articles in group (*a*) include [192,194,204,210,214,221,222,223]. Therefore, in the earliest of them, chronologically [192], a detective analysis of the radial distribution in the exchange energy, hinting at ideas about new types of density functionals, was dedicated to the specifics of the electronic structure of atoms, exploiting the intrinsic spherical symmetry. In [204], the authors made a systematic study of partial fourth-order perturbative schemes due to triples, to compute the ionization potential within the Fock space multi-reference coupled-cluster theory. In particular, we have obtained computationally less expensive correlation schemes due to fourth-order triples. In [214], the fashionable Parr–Pearson (PP) atoms-in-molecule/bonding (AIM/AIB) approach, for determining the exchanged charge necessary for acquiring an equalized electronegativity within a chemical bond, was refined and generalized by introducing the concepts of chemical power within the chemical orthogonal space (COS), in terms of electronegativity and chemical hardness. It is noteworthy that the articles in group (*a*) include 3 review articles out of 4, related to this thematic area, namely [194,222,223]. In the first of them, chronologically [194], a review of the development and application of the multipolar and polarizable force field AMOEBA for ionic liquid systems, termed AMOEBA–IL, was presented. The second review article [222], focused on a combination of ab initio molecular dynamics (aiMD) and NMR parameter calculations using quantum mechanical methods. The third one [223], was devoted to the problem of the prognostication and design of functional and material properties of nano-catalysts and drugs. The authors of this review, in particular, considered the data handling and sharing problems in the design and discovery of catalyst candidates, particularly material informatics and catalyst design, structural coding, data collection and validation, infrastructure for catalyst design, and the online databases for catalyst design.

In accordance with the title of the given Journal, however, one would expect that most publications in this thematic area should be articles in which the subject of research is the quantum–chemical calculation of any quantitative parameters of molecules with chemical compounds associated with their spatial structure, absorption spectra, and magnetism, as well as with the reaction mechanisms of their formation. This expectation is fully consistent with reality, since most of the articles in this direction (31 of 39) are precisely the articles in groups (*b*) and (*c*). Moreover, the articles in group (*b*) constitute the largest part of all the articles related to Thematic Area 6; these include [35,134,190,191,196,197,198,201,203,205,206,207,211,216,217,218,219]. For formal reasons, the list of just cited references can also include the original article [208] and the review [220] devoted to graphene and fullerenes, since they all contain carbon–carbon bonds, and, according to the IUPAC standards, they should be classified as organic compounds (although, in general, this means that all chemical compounds consisting of atoms of only one chemical element are traditionally considered objects of inorganic chemistry). Some of these articles are about biologically important organic compounds; these include [134,198,203,207,216,217]. The most interesting of them, in our opinion, is the work [216], in which, for studying the structure; electron affinity; populations via Boltzmann distribution; and one-electron reduction potentials (E°) of 2′-deoxyribose sugar radicals in the aqueous phase, the DFT B97XD/6-31++G** basis set was used, wherein 2′-deoxyguanosine and 2′-deoxythymidine were considered as models of DNA. According to the results of this calculation, the relative stability of sugar radicals is changed in the order C4′• > C1′• > C5′• > C3′• > C2′•. Among other articles from the same group, article [197] should be noted, in which the object of research was the Diels–Alder reaction. In this research, the given reaction between cyclopentadiene and methyl acrylate was computationally investigated in the ionic liquid 1-butyl-3-methylimidazolium hexafluorophosphate, [BMIM] [PF_6_], as a basis for the validation of the OPLS-VSIL to properly reproduce a reaction medium environment. The articles of group (*c*) include publications [28,129,136,193,195,199,200,202,208,209,212,213,215,220]: in most of them [129,136,195,199,200,202,208,209,212,215], the objects of quantum chemical calculations were the chelate and macrocyclic coordination compounds of various *s*-, *p*-, *d*-, and *f*-elements. The most significant of them seems to be publication [136], devoted to Pu(IV) and Pu(VI) complexes with tridentate (NNN)-donor-atomic ligands, in particular, di(pyridyl-2)amine, 1,3-diamino-2-azapropane, and 2,6-di(pyridyl-2) pyridine, the results of which can be very useful in the development of new extractants and organic sensors of plutonium. In [129,213], quantum–chemical methods used for calculating DFT OPBE/TZVP and DFT B3PW91/TZVP were used to substantiate the fundamental possibility of the existence of complexes of iron, cobalt, and nickel with porphyrazine, its derivatives, and oxo ligands in oxidation state VI that is unusual for these elements. Inorganic compounds that are not coordination compounds were considered only in [28,193,215,220]. It should be noted that the only review in this thematic area is [220], devoted specifically to the quantum–chemical calculations of chemical compounds, namely, the structures of higher fullerenes, in which, among other things, a formalization of the substructure approach to assessing the stability of higher fullerenes is proposed, which is based on a detailed analysis of the main structural features of fullerene molecules. Furthermore, article [193] is noteworthy, which considered the adsorption behaviors of CO and NO, and the reaction of NO reduction with CO on the ZrO_2_ (110) and (111) surfaces was performed through periodic density functional theory (DFT) calculations. It is interesting to note that namely, in this group of articles, 2 articles out of 5 of the Editorial type [199,215] are presented, which are included in the general list of 300 articles considered by us in the given our review.

### 2.7. The Structure and Reactivity of Gas-Phase Ions and Radicals

Within the framework of this direction, only 11 articles have been published [224,225,226,227,228,229,230,231,232,233,234], among which there is 1 review. In terms of its subject matter, this direction, to some extent, intersects with both Thematic Area 5 and Thematic Area 6, since ions and radicals are among the objects of Thematic Area 5, and the structure and reactivity of these compounds, obtained as a result of theoretical research (carried out, as a rule, by quantum–chemical methods), are among the objects of Thematic Area 6. For the sake of fairness in this regard, it is worth noting that the assignment of the 11 articles mentioned above to Thematic Area 7 cannot be considered unambiguous, although, in our opinion, their assignment to this direction is the most justified. The distribution of these articles in accordance with the above typification is given in Table 8. By analogy, with the systematization of articles indicated in Thematic Area 5, here, it is also advisable to subdivide all publications presented in it into two groups, in the first of which there will be articles on organic compounds, in the second, articles on inorganic compounds. Following this differentiation, the first of these groups should include 7 articles, namely [227,228,229,230,231,232,234], and the second, 4 articles [224,225,226,233]. The most significant among them, in our opinion, is review [231], devoted to the physical chemistry of one of the most stable known radicals, 2,2-diphenyl-1-(2,4,6-trinitrophenyl)hydrazyl (also called diphenylpycrylhydrazil or DPPH), which was opened in 1992, and will make it exactly 100 years old, in 2022. The rest of the articles of this thematic direction, the objects of which were organic compounds, for the most part, were also related to the study of radical particles; for example, in [230], this was the azide radical, in [228,229], the radicals formed as a result of the interaction of 5-nitro-2-chloropyrimidine and 2-nitrofuran, respectively, with a flux of low-energy electrons. The only exception here is paper [232], in which the object of study was the C_6_H_6_^+^ cation formed during the ionization of benzene as a result of electron impact. A similar situation with the number of publications devoted to radicals and ions takes place in the case of inorganic substances, where in three out of four articles, the objects of study were radicals, and, only in one [226], anions were formed by various fullerenes. In this regard, we would like to note publications [225,233], in the first of which the determination of the nitroxyl radical was carried out using 4-amino-2,2,6,6-tetramethylpiperidinyl-N-oxyl, and, in the second, the specificity of the reaction of the hydroxyl radical with methionine (Met) residues in peptides and proteins in a biomimetic model system consisting of Ac-Met-OMe was used as the simplest model peptide backbone. This thematic direction undoubtedly needs significant replenishment with new publications.

### 2.8. Energy/Charge Transfer Dynamics in Organic/Inorganic Materials

We assigned 18 articles to this thematic area, namely [128,235,236,237,238,239,240,241,242,243,244,245,246,247,248,249,250,251]. Their distribution within the typing specified in the Introduction is presented in Table 9. As in most of the previous thematic sections, in Thematic Area 8, only original research and review articles are presented; since the title of this area contains the phrase “organic/inorganic materials”, it makes sense to systematize the articles included in it using the same approach as when considering publications of Thematic Areas 5 and 7. Taking this circumstance into account, two basic variants of their systematics are possible—by the nature of the transfer (energy transfer or charge transfer) and by the physicochemical nature of the material, in which such a transfer is carried out. In the variant of systematics by the nature of transfer, all articles presented in this Thematic Area can be divided into those in which studies are related to energy transfer, and those in which studies are related to charge transfer; the first include articles [128,236,237,238,239,241,242,246,247,249], the second, articles [235,240,243,244,245,248,250,251]. In the variant of systematics by the nature of the material, two types of articles can also be distinguished—those in which the energy/charge transfer is carried out in organic materials, namely [128,236,237,238,240,243,244,247,249,250,251], and those in which this transfer is carried out in inorganic materials [235,239,241,242,245,246,248]. The second variant of the systematics of articles seems to be more expedient, which we will use as the basis for further narration about Thematic Area 8. As it should be expected, a priori, most of the articles in the direction of the PCCP Section of *IJMS* should be related to organic materials, and this turns out to be the case, since these include 11 out of 18 articles related to this Thematic Area. Speaking about them, we can note that in the earliest of them, chronologically [236], the energetics of the reversible phase transfer of various ionic liquid surfactants—derivatives based on azobenzene between the organic and aqueous phases were investigated and the key factors affecting the efficiency of this process were identified. In [236], the discussion was about the transfer of energy; an example of an article in which the study of charge transfer took place is [240], in which the intramolecular charge transfer in a probe based on benzothiazole was recorded. These two articles, as well as [128,244,251], were devoted to organic abiogenic materials; in other articles of the “organic” profile, namely [237,238,243,247,249,250], materials of biological origin, very diverse in terms of their physicochemical characteristics, were used as a medium for the transfer of energy/charge—polypeptides and their derivatives [237,243,250], nitrogenase [247], and various antibiotics [249]. Among the research articles related to the above processes in inorganic materials, it should be noted that [241] was devoted to the creation of quantum dots from carbon atoms in an array of tricarbon tetranitride C_3_N_4_, and article [246] demonstrated the expediency of short-term sonoporation using microbubbles encapsulated by nanoparticles elemental gold in the treatment of liver cancer before radiation therapy. Within the framework of this Thematic Area, during the reporting period, three review articles were also published [238,242,247], each of which deals with the processes associated with the transfer of energy; moreover, in the first and third of them, this transfer occurs in organic materials, in the second, in the inorganic materials.

### 2.9. Physical Processes in Nanomaterials

In this Thematic Area, 56 articles have been published [48,64,104,108,110,124,195,252,253,254,255,256,257,258,259,260,261,262,263,264,265,266,267,268,269,270,271,272,273,274,275,276,277,278,279,280,281,282,283,284,285,286,287,288,289,290,291,292,293,294,295,296,297,298,299,300], and all 4 of the above categories are represented here by at least 1 article (Table 10). It is possible to subdivide these articles by topic in the same way as in the previous Thematic Area (8), namely, with an emphasis on which of the two basic types of taxonomy of chemical substances—organic or inorganic—the material in which the corresponding physical process is realized belongs. A more detailed examination shows that, in this direction, as well as in the previous ones, articles in which the objects of research are organic materials clearly prevail over articles in which the objects of research are inorganic materials (36 and 20, respectively). Wherein, the overwhelming majority of articles of the “organic” profile are, in one way or another, connected with the participation of the objects with a biological origin; these include [64,124,254,255,256,257,258,259,260,261,262,263,274,277,278,282,284,289,291,292,293,294,295,296,298,299,300] and the processes in abiogenic organic materials were considered only in [48,195,266,271,272,273,283,288,290]. The thematic focus of these works, as well as the chemical compounds studied in them, is also very diverse, and even a simple listing of them here is clearly beyond the scope of this article, too. So, in [64], the effect of cholesterol on the structure, mechanics, and water permeability of an amphiphile bilayer with an ionic pair (IPA) of hexadecyltrimethyl-ammonium dodecyl sulfate and dodecyltrimethyl-ammonium hexadecyl sulfate was shown to cause the ordering of alkyl chains around the rigid sterol ring, and increases the cavity density in the hydrophilic region of both IPA bilayers. In [259], it was proposed to use plant suspension-cultured cells (PSCC) with cyclodextrins (CD) as an alternative method for the production of bioactive compounds from the *Bryophyllum* species. In [271], a three-symmetric trioxotriangulene derivative with three pyridyl groups as coordination centers was synthesized, which, according to the cyclic voltammetry data, showed a multistep redox ability from neutral radical to radical tetraanionic forms. The physical and physicochemical processes in organic materials are also devoted to 9 out of 11 review articles belonging to Thematic Area 9, namely [254,261,274,289,291,293,298]; all of these reviews are, in one way or another, related to biological systems. It is worth noting, however, a review article [274] that is devoted to such an extremely urgent problem today, the fight against the “plague of the XXI century”, the coronavirus infection (COVID-19), in particular the timely detection of this disease and the development of methods for its treatment using nanomaterials and/or nanotechnology. In our opinion, the data of the review [291] devoted to the physic–chemistry of bile acids in various materials (including materials with a nanostructural level of organization) can also be of considerable interest.

The processes in inorganic nanomaterials were considered in one way or another, in articles [104,108,110,252,253,264,265,267,268,269,270,275,276,279,281,285,286,287,297]. Additionally, here, the materials in which these processes are implemented are very diverse. Article [252] is particularly worth noting, which describes such a unique object as a rotary nanomotor, consisting of wedge-shaped diamonds and triple nanotubes, in which the so-called outer and middle nanotubes were used as a kind of “nano-bearing” to restrain the inner tube. The authors of [265] proposed a very fast and, at the same time, economical and environmentally friendly technology of microwave synthesis in one vessel for the preparation of ternary nanocomposites carbon/polydopamine/Au nanoparticles. It is remarkable and important that this technology is not associated with high-temperature processes and can be implemented under standard conditions.

Using the method of sol-gel technologies in [270], multifunctional hybrid materials were based on silica matrices containing inclusions or combinations of elemental platinum nanoparticles + 5,10,15,20-tetra(4-allyloxyphenyl)porphyrin, or nanoparticles of the Pt(II) complex with this macrocyclic ligand. Concluding this brief review, we note that among the articles of the “inorganic” profile there are also two reviews [269,281], the first of which is devoted to the analysis of the literature related to photodynamic therapy with the participation of nanoparticles and the prospects for its use for the treatment of oncological diseases, the second, to physical processes in nanomaterials based on mesoporous silicon dioxide particles.

## 3. Articles in Special Issues

Since 2019, in the PCCP section of *IJMS*, the so-called Special Issues, which, following their titles, published articles on advances in a relatively narrow field of the branch of molecular science. For each such issue, the Guest Editor was handpicked from a number of scientists well known in the industry. The first release, entitled “Ion and Molecule Transport in Membrane Systems”, was organized at the end of 2018; its Guest Editor was Professor Victor V. Nikonenko, and the first article in this issue [59] was published on 4 January 2019. It is noteworthy that this was a review article that was devoted to profiled membranes (also known as corrugated membranes, micro-structured membranes, patterned membranes, membranes with designed topography, or notched membranes) that are gaining increasing academic and industrial attention, and recognition as a viable alternative to flat membranes.

It should be noted that in this connection, the given issue turned out to be a kind of “record holder” in terms of the number of articles published in it (22) among all the Special Issues organized in *IJMS*. (In this regard, its sequel was even organized, namely “Ion and Molecule Transport in Membrane Systems 2.0”, which included 19 more articles). Moreover, it is worth noting that most of the 300 articles we consider, are from a Special Issue, while, in Thematic Area 1, there are 18, which is 72.0% of their total number (25); in Thematic Area 2, 16 out of 22 (72.7%); in Thematic Area 3, 6 out of 12 (50.0%); in Thematic Area 4, 49 out of 50 (98.0%); in Thematic Area 5, 67 out of 91 (73.6%); in Thematic Area 6, 28 out of 39 (71.8%); in Thematic Area 7, 8 out of 11 (72.7%); in Thematic Area 8, 12 out of 18 (66.7%); and in Thematic Area 9, 44 out of 55 (80.0%). As it can be seen from these numbers, not only in the Section as a whole, but in each of these Thematic Areas, at least half of the articles presented are included in Special Issues, and in Thematic Area 4, almost all articles. (In this regard, it is noteworthy that in five of these directions, namely 1, 2, 5, 6, and 7, the above values are very close to each other and are in a rather narrow range, namely 71–74%). Of the 300 articles considered in this review, 254 generally belong to Special Issues, i.e., more than 80% of their total. Due to the very significant thematic diversity of these Special Issues, it is most expedient to systematize them in the areas to which the articles published in them belong. The number of Special Issues, which include articles related to a particular Thematic Area (1–9), for each of these areas is as follows:

Thematic Area 1—articles from **11** various Special Issues,

Thematic Area 2—articles from **5** various Special Issues,

Thematic Area 3—articles from **5** various Special Issues,

Thematic Area 4—articles from **6** various Special Issues,

Thematic Area 5—articles from **19** various Special Issues,

Thematic Area 6—articles from **17** various Special Issues,

Thematic Area 7—articles from **5** various Special Issues,

Thematic Area 8—articles from **8** various Special Issues,

Thematic Area 9—articles from **22** various Special Issues.

### 3.1. Completed Special Issues

At the beginning of December 2021, 24 Special Issues were completed within the PCCP section, of which 15 were completed during the period considered in this review, from October 2018 to May 2021; details of these releases of *IJMS* can be found on the website https://www.mdpi.com/journal/IJMS/special_issues?section_id=0&search=&sort=deadline&view=closed&page_count=50 (accessed on 5 December 2021).

These 15 Special Issues include 170 articles. Moreover, in each of the above 9 Thematic Areas, articles are presented related to different issues; the titles of these Special Issues for each of these Thematic Areas (in alphabetical order) are presented below.

Thematic Area 1—**5** Special Issues:

♦ Assembly Superstructures in Chemistry;
♦ Conformations and Physicochemical Properties of Biological Ligands in Various Environments;
♦ Host–Guest Complexes;
♦ Molecular Structure and Dynamics Probed by Spectroscopic Techniques and Computational Approaches: New Trends by NMR, FTIR, Neutron Scattering and Simulation;
♦ Ordered Mesoporous Materials;

Thematic Area 2—**4** Special Issues:

♦ Advances in the Chemistry of Porphyrins and Related Macrocycles;
♦ Assembly Superstructures in Chemistry;
♦ Ion and Molecule Transport in Membrane Systems;
♦ Solution Chemical Kinetics 2.0;

Thematic Area 3—**2** Special Issues:

♦ Ion and Molecule Transport in Membrane Systems;
♦ Molecular and Ionic Dynamics by Means of Nuclear Magnetic Resonance;

Thematic Area 4—**4** Special Issues:

♦ Assembly Superstructures in Chemistry;
♦ Ion and Molecule Transport in Membrane Systems;
♦ Ion and Molecule Transport in Membrane Systems 2.0;
♦ Ordered Mesoporous Materials;

Thematic Area 5—**10** Special Issues;

♦ Advanced Photochemistry and Photobiology within Confining Chemical and Biological Hosts;
♦ Assembly Superstructures in Chemistry;
♦ Conformations and Physicochemical Properties of Biological Ligands in Various Environments;
♦ Host–Guest Complexes;
♦ Molecular Structure and Dynamics Probed by Spectroscopic Techniques and Computational Approaches: New Trends by NMR, FTIR, Neutron Scattering and Simulation;
♦ Nanoparticle-Based Radiosensitization 2.0;
♦ Oligonuclear Metal Complexes with Schiff Base Ligands;
♦ Quantum–Chemical Modeling and Design of Chelate and Macrocyclic Metal Complexes;
♦ Solution Chemical Kinetics 2.0;
♦ Synthesis and Reactivity of Novel Aromatic Compounds;

Thematic Area 6—**6** Special Issues:

♦ Host-Guest Complexes;
♦ Molecular and Ionic Dynamics by Means of Nuclear Magnetic Resonance;
♦ Molecular Structure and Dynamics Probed by Spectroscopic Techniques and Computational Approaches: New Trends by NMR, FTIR, Neutron Scattering and Simulation;
♦ Quantum–Chemical Modeling and Design of Chelate and Macrocyclic Metal Complexes;
♦ Solution Chemical Kinetics 2.0;
♦ Synthesis and Reactivity of Novel Aromatic Compounds;

Thematic Area 7—**1** Special Issue:

♦ Synthesis and Reactivity of Novel Aromatic Compounds;

Thematic Area 8—**3** Special Issues:

♦ Molecular Structure and Dynamics Probed by Spectroscopic Techniques and Computational Approaches: New Trends by NMR, FTIR, Neutron Scattering and Simulation;
♦ Solution Chemical Kinetics 2.0;
♦ Synthesis and Reactivity of Novel Aromatic Compounds;

Thematic Area 9—**9** Special Issues

♦ Advances in the Chemistry of Porphyrins and Related Macrocycles;
♦ Assembly Superstructures in Chemistry;
♦ Conformations and Physicochemical Properties of Biological Ligands in Various Environments;
♦ Host–Guest Complexes;
♦ Molecular Structure and Dynamics Probed by Spectroscopic Techniques and Computational Approaches: New Trends by NMR, FTIR, Neutron Scattering and Simulation;
♦ Ion and Molecule Transport in Membrane Systems;
♦ Nanoparticle-Based Radiosensitization 2.0;
♦ Ordered Mesoporous Materials;
♦ Synthesis and Reactivity of Novel Aromatic Compounds.

### 3.2. Continued Special Issues after 31 May 2021

The full list of Special Issues within the PCCP Section, continuing after 31 May 2021, in each of which at least 1 out of the 300 articles was published, we were considering, including 28 titles. Some of them (9) were completed in the period from 1 June to 1 December 2021, while the rest (19) are scheduled for completion in 2022; details of these releases in *IJMS* can be found on the website https://www.mdpi.com/journal/IJMS/special_issues?section_id=447&search=&sort=deadline&view=open&page_count=50 (accessed on 5 December 2021).

These special editions include 84 articles from the above list of 300 articles. The titles of these Special Issues for each of these Thematic Areas (in alphabetical order) are shown below.

Thematic Area 1—**6** Special Issues:

❖ Advances in Molecular Simulation;
❖ Food Hydrocolloids: Molecular Vision;
❖ From Molecules to Colloids: Recent Advances in Their Chemical Physics;
❖ Ionic Liquids: Applications in Energy and Environment;
❖ Kinetic Manifestation of Bimolecular Multistage Physicochemical Processes in Solutions;
❖ The Structure and Function of the Second Phase of Liquid Water;

Thematic Area 2—**1** Special Issue:

❖ The Structure and Function of the Second Phase of Liquid Water;

Thematic Area 3—**3** Special Issues:

❖ Applications in Energy and Environment;
❖ Electrocatalysts for Electrochemical Sensors and Energy-Related Devices;
❖ Ionic Liquids: Applications in Energy and Environment;

Thematic Area 4—**2** Special Issues:

❖ Future Trends in Biomaterials and Devices for Cells and Tissues;
❖ Nanoparticle-Based Radiosensitization 2.0;

Thematic Area 5—**9** Special Issues;

❖ Advances in the Chemistry of Porphyrins and Related Macrocycles;
❖ Advances in Molecular Simulation;
❖ Computational Modeling of Ionic Liquids and Solutions for Modern Applications;
❖ Feature Papers in Physical Chemistry and Chemical Physics;
❖ From Molecules to Colloids: Recent Advances in Their Chemical Physics;
❖ Nanomaterials in Cancer Diagnosis and Therapy;
❖ NIR and SWIR Contrast Agents for Theragnostic Applications;
❖ Recent Development of Organic Field-Effect Transistors Based on Molecule Structure;
❖ The Structure and Function of the Second Phase of Liquid Water;

Thematic Area 6—**10** Special Issues:

❖ Advances in Chemical Bond and Bonding;
❖ Advances in Molecular Simulation;
❖ Computational Modeling of Ionic Liquids and Solutions for Modern Applications;
❖ Feature Papers in Physical Chemistry and Chemical Physics;
❖ From Molecules to Colloids: Recent Advances in Their Chemical Physics;
❖ Molecular Nano-Architectures: Chemistry and Physics;
❖ New Hybrid Materials for Nonlinear Optics;
❖ Physical Analysis of Nanomaterials;
❖ Radiation-Induced Damage to DNA 2.0;
❖ Simple Substances of Non-Metals: Molecular Structures Modeling with Using DFT and More Advanced Methods of Quantum Chemistry;
❖ Ultrafast Non-Adiabatic Processes in Molecules: Recent Advances in Theory and Experiment;

Thematic Area 7—**4** Special Issues:

❖ Electron and Photon Interactions with Bio(Related) Molecules;
❖ Feature Papers in Physical Chemistry and Chemical Physics;
❖ Radiation and Photochemical Modifications in Proteins: Mechanistic Aspects and Applications);
❖ Radiation-Induced Damage to DNA 2.0;

Thematic Area 8—**5** Special Issues:

❖ Electron and Photon Interactions with Bio(Related) Molecules);
❖ Feature Papers in Physical Chemistry and Chemical Physics;
❖ Kinetic Manifestation of Bimolecular Multistage Physicochemical Processes in Solutions;
❖ Molecular Research Approaches for Energy Storage Systems beyond Lithium;
❖ Nanoparticle-Based Radiosensitization 2.0;

Thematic Area 9—**13** Special Issues

Advanced Photochemistry and Photobiology within Confining Chemical and Biological Hosts;

❖ Electrocatalysts for Electrochemical Sensors and Energy-Related Devices;
❖ Electron and Photon Interactions with Bio(Related) Molecules;
❖ Feature Papers in Physical Chemistry and Chemical Physics;
❖ Food Hydrocolloids: Molecular Vision;
❖ Future Trends in Biomaterials and Devices for Cells and Tissues;
❖ Molecular Nano-Architectures: Chemistry and Physics;
❖ Molecular Research Approaches for Energy Storage Systems beyond Lithium;
❖ Nanoparticle-Based Radiosensitization 2.0;
❖ Nanoparticles for Tumor Imaging and Therapy;
❖ New Hybrid Materials for Nonlinear Optics;
❖ Physical Analysis of Nanomaterials;
❖ Radiation-Induced Damage to DNA 2.0.

In general, according to the author of these lines, such an editorial policy with an emphasis on placing articles published in the journal within the framework of such issues has every right to exist and has unequivocally proved its appropriateness.

## 4. Conclusions

As can be seen from the above, the topics of the first 300 articles published in the *International Journal of Molecular Sciences* under the auspices of the *Physical Chemistry and Chemical Physics* Section are very thematic in diversity. This diversity, however, does not seem to be something unusual, since both the title of this Section and the title of the Journal itself cover a very wide range of physicochemical processes, as well as related research objects. Nevertheless, we would like to draw attention to the following important points.

Firstly, the number of articles both in the *International Journal of Molecular Sciences* and in the *Physical Chemistry and Chemical Physics* Section is growing from year to year, and at a very rapid pace (from 3 during 2018, to 186 during 2021, and to 417 in total at the beginning of December 2021). What is characteristic, despite the constant annual increase in the number of published articles in *IJMS*, is that its impact factors are also growing in each of the two main international citation databases—both in the Web of Science and in Scopus, and this circumstance, as the author of this review believes, should delight both the Editors of the Journal and those scientists who have already published their articles in it or intend to do so in the future.

Secondly, a very significant part of the works published in the PCCP Section of *IJMS* for the first three-year period, is devoted to the consideration of certain problems associated with the so-called Life Sciences, in particular various aspects of molecular biology, biosynthesis, and medicine. The role of physical chemistry proper and chemical physics in these works was rather secondary and, in fact, was reduced only to the use of various physical physicochemical methods of analysis and quantum–chemical methods of calculation for solving the corresponding problems indicated in these articles. As a result, a considerable part of the articles in this Section should be attributed, rather, to the field of biochemistry and/or medicine, and in general to “life science”, rather than to the field of physical chemistry or chemical physics. This somewhat unusual situation becomes clear when you consider that, according to an excerpt from the official website of the Editorial Office (https://www.mdpi.com/journal/IJMS (accessed on 5 December 2021): “*International Journal of Molecular Sciences* is an international, peer-reviewed, open access journal providing an advanced forum for biochemistry, molecular and cell biology, molecular biophysics, molecular medicine, and all aspects of molecular research in chemistry, and is published semimonthly online by MDPI”. With this in mind, it is quite obvious that most of the articles in the *IJMS* as a whole should be represented precisely by articles related to the Life Sciences. However, the PCCP Section is not directly related to the Life Sciences in thematic respect, and, probably, when attributing articles published in *IJMS* to this section, this circumstance should be taken into account and, mainly, articles related to the field of physical inorganic or physical organic chemistry, and not to the field of Life Sciences.

Thirdly, and this is to a large extent directly related to what was said above, our assignment of individual articles (at least some of them) to one or another of the above nine “canonical” thematic areas in a number of cases is rather arbitrary. It is possible that not all readers of this review article can agree with their subdivision, according to the sections officially accepted in the PCCP, which was performed in the given article. However, be that as it may, the Journal is improving from year to year, and the circumstance of the author of this article (and, at the same time, the Member of the Editorial Board of the PCCP Section) is very, very happy. Furthermore, we very much hope that this positive trend will continue in the future, too.

## Figures and Tables

**Table 1:** The number of articles of various levels published in *IJMS* during the period October 2018–May 2021 (total 300 articles).

Year	Number of Published Articles				
	Reviews	Original	*Communications*	Editorials	Total
2018	**0**	3	*0*	0	**3**
2019	**6**	65	*2*	0	**73**
2020	**20**	131	*1*	2	**154**
2021	**14**	52	*1*	3	**70**
Total	**40**	251	*4*	5	**300**

**Table 2:** The number of articles of various levels published in the framework of Thematic Area 1 during the period October 2018–May 2021.

Year	Number of Published Articles				
	Reviews	Original	*Communications*	Editorials	Total
2018	**0**	1	*0*	0	**1**
2019	**0**	3	*0*	0	**4**
2020	**2**	15	*0*	0	**16**
2021	**1**	3	*0*	0	**4**
Total	**3**	22	*0*	0	**25**

**Table 3:** The number of articles of various levels published in the framework of Thematic Area 2 during the period October 2018–May 2021.

Year	Number of Published Articles				
	Reviews	Original	*Communications*	Editorials	Total
2018	**0**	0	*0*	0	**0**
2019	**0**	11	*0*	0	**11**
2020	**0**	9	*0*	0	**9**
2021	**0**	2	*0*	0	**2**
Total	**0**	22	*0*	0	**22**

**Table 4:** The number of articles of various levels published in the framework of Thematic Area 3 during the period October 2018–May 2021.

Year	Number of Published Articles				
	Reviews	Original	*Communications*	Editorials	Total
2018	**0**	1	*0*	0	**1**
2019	**0**	2	*0*	0	**2**
2020	**2**	3	*0*	0	**5**
2021	**0**	3	*0*	0	**3**
Total	**2**	9	*0*	0	**11**

**Table 5:** The number of articles of various levels published in the framework of Thematic Area 4 during the period October 2018–May 2021.

Year	Number of Published Articles				
	Reviews	Original	*Communications*	Editorials	Total
2018	**0**	0	*0*	0	**0**
2019	**2**	16	*1*	0	**19**
2020	**3**	20	*0*	0	**23**
2021	**0**	6	*0*	2	**8**
Total	**5**	42	*1*	2	**50**

**Table 6:** The number of articles of various levels published in the framework of Thematic Area 5 during the period October 2018–May 2021.

Year	Number of Published Articles				
	Reviews	Original	*Communications*	Editorials	Total
2018	**0**	2	*0*	0	**2**
2019	**1**	18	*1*	0	**20**
2020	**7**	46	*1*	0	**54**
2021	**3**	12	*0*	0	**15**
Total	**11**	78	*2*	0	**91**

**Table 7:** The number of articles of various levels published in the framework of Thematic Area 6 during the period October 2018–May 2021.

Year	Number of Published Articles				
	Reviews	Original	*Communications*	Editorials	Total
2018	**0**	0	*0*	0	**0**
2019	**0**	6	*0*	0	**6**
2020	**1**	21	*0*	1	**23**
2021	**3**	6	*0*	1	**10**
Total	**4**	33	*0*	2	**39**

**Table 8:** The number of articles of various levels published in the framework of Thematic Area 7 during the period October 2018–May 2021.

Year	Number of Published Articles				
	Reviews	Original	*Communications*	Editorials	Total
2018	**0**	0	*0*	0	**0**
2019	**0**	2	*0*	0	**2**
2020	**0**	4	*0*	0	**4**
2021	**1**	4	*0*	0	**5**
Total	**1**	10	*0*	0	**11**

**Table 9:** The number of articles of various levels published in the framework of Thematic Area 8 during the period October 2018–May 2021.

Year	Number of Published Articles				
	Reviews	Original	*Communications*	Editorials	Total
2018	**0**	0	*0*	0	**0**
2019	**1**	5	*0*	0	**6**
2020	**1**	6	*0*	0	**7**
2021	**1**	4	*0*	0	**5**
Total	**3**	15	*0*	0	**18**

**Table 10:** The number of articles of various levels published in the framework of Thematic Area 9 during the period October 2018–May 2021.

Year	Number of Published Articles				
	Reviews	Original	*Communications*	Editorials	Total
2018	**0**	1	*0*	0	**1**
2019	**2**	12	*0*	0	**14**
2020	**5**	17	*0*	1	**23**
2021	**4**	13	*1*	0	**18**
Total	**11**	43	*1*	1	**56**

## Data Availability

The given study did not report any data.

## Supplementary Materials

Supplementary materials can be found at https://www.mdpi.com/article/10.3390/ijms23010241/s1.
