# Supplementary material for: The Physical Chemistry and Chemical Physics (PCCP) Section of the International Journal of Molecular Sciences in Its Publications: The First 300 Thematic Articles in the First 3 Years
Source: Int J Mol Sci. 2021 Dec 27;23(1):241. doi: 10.3390/ijms23010241 (PMC8745423; doi:10.3390/ijms23010241)
Supplement: Supplementary file 1 [file ijms-23-00241-s001.zip › IJMS2021_3_Supplementary material 1.pdf]

International Journal of *Molecular Sciences*

MDPI

Supplementary Material 1

First 300 articles published in Physical Chemistry & Chemical Physics section of *International Journal of Molecular Science* listed in chronological order

1. Zonglin Liu, Dongfeng Wang, Xun Sun, Qingjie Sun, Yanjiang Wu and Ying Xu, Construction and Characterization of Phthalocyanine-Loaded Particles of Curdlan and Their Photosensitivity. *Int. J. Mol. Sci.* **2018**, *19*, 3323; https://doi.org/10.3390/ijms19113323 (Ref. **48** in the References list to the given article)

2. Poonam Mudgil, Douglas Borchman and Aparna Ramasubramanian, Insights into Tear Film Stability from Babies and Young Adults: A Study of Human Meibum Lipid Conformation and Rheology. *Int. J. Mol. Sci.* **2018**, *19*, 3502; https://doi.org/10.3390/ijms19113502 (Ref. **1** in the References list to the given article)

3. Kazuki Yatabe, Masaru Hisada, Yudai Tabuchi and Masumi Taki, A Cysteine-Reactive Small Photo-Crosslinker Possessing Caged-Fluorescence Properties: Binding-Site Determination of a Combinatorially-Selected Peptide by Fluorescence Imaging/Tandem Mass Spectrometry. *Int. J. Mol. Sci.* **2018**, *19*, 3682; https://doi.org/10.3390/ijms19113682 (Ref. **102** in the References list to the given article)

4. Sylwin Pawlowski, João G. Crespo and Svetlozar Velizarov. Profiled Ion Exchange Membranes: A Comprehensible Review. *Int. J. Mol. Sci.* **2019**, *20*, 165; https://doi.org/10.3390/ijms20010165 (Ref. **59** in the References list to the given article)

5. Guillaume Dufton, Sergey Mikhaylin, Sami Gaaloul and Laurent Bazinet. Positive Impact of Pulsed Electric Field on Lactic Acid Removal, Demineralization and Membrane Scaling during Acid Whey Electrodialysis. *Int. J. Mol. Sci.* **2019**, *20*, 797; https://doi.org/10.3390/ijms20040797 (Ref. **60** in the References list to the given article)

6. Sui-Ping Deng, Yi-Li Yang, Xing-Xing Cheng, Wen-Rong Li and Ji-Ye Cai. Synthesis, Spectroscopic Study and Radical Scavenging Activity of Kaempferol Derivatives: Enhanced Water Solubility and Antioxidant Activity. *Int. J. Mol. Sci.* **2019**, *20*, 975; https://doi.org/10.3390/ijms20040975 (Ref. **103** in the References list to the given article)

7. Aaron Byrne, Eduardo M. Bringa, Mario G. Del Pópolo, Jorge J. Kohanoff, Vanesa Galassi and Niall J. English. Mechanisms of Iodide–Triiodide Exchange Reactions in Ionic Liquids: A Reactive Molecular-Dynamics Exploration. *Int. J. Mol. Sci.* **2019**, *20*, 1123; https://doi.org/10.3390/ijms20051123 (Ref. **235** in the References list to the given article)

8. Hui Li, Aiqin Wang, Jiao Shi,Yongjian Liu and Gao Cheng. Diamond Needles Actuating Triple-Walled Carbon Nanotube to Rotate via Thermal Vibration-Induced Collision. *Int. J. Mol. Sci.* **2019**, *20*, 1140; https://doi.org/10.3390/ijms20051140 (Ref. **252** in the References list to the given article)

9. Quan-De Wang, Mao-Mao Sun and Jin-Hu Liang. Reaction Mechanisms and Kinetics of the Hydrogen Abstraction Reactions of C4–C6 Alkenes with Hydroxyl Radical: A Theoretical Exploration. *Int. J. Mol. Sci.* **2019**, *20*, 1275; https://doi.org/10.3390/ijms20061275 (Ref. **190** in the References list to the given article)

10. Maria Rosaria di Nunzio, Ganchimeg Perenlei and Abderrazzak Douhal. Confinement Effect of Micro- and Mesoporous Materials on the Spectroscopy and Dynamics of a Stilbene Derivative Dye. *Int. J. Mol. Sci.* **2019**, *20*, 1316; https://doi.org/10.3390/ijms20061316 (Ref. **104** in the References list to the given article)

11. Tatiana Yakhno, Mikhail Drozdov and Vladimir Yakhno Giant Water Clusters: Where Are They From? *Int. J. Mol. Sci.* **2019**, *20*, 1582; https://doi.org/10.3390/ijms20071582 (Ref. **105** in the References list to the given article)

12. Zhiyong Li, Ying Feng, Xiaoqing Yuan, Huiyong Wang, Yuling Zhao and Jianji Wang. Photo-Triggered Reversible Phase Transfer of Azobenzene-Based Ionic Liquid Surfactants between Oil and Water. *Int. J. Mol. Sci.* **2019**, *20*, 1685; https://doi.org/10.3390/ijms20071685 (Ref. **236** in the References list to the given article)

13. Giuseppe Battaglia, Luigi Gurreri, Girolama Airò Farulla, Andrea Cipollina, Antonina Pirrotta, Giorgio Micale and Michele Ciofalo. Membrane Deformation and Its Effects on Flow and Mass Transfer in the Electromembrane Processes. *Int. J. Mol. Sci.* **2019**, *20*, 1840; https://doi.org/10.3390/ijms20081840 (Ref. **61** in the References list to the given article)

14. Hongjing Han, Jinxin Li, Qin Ge, Yizhen Wang, Yanguang Chen and Baohui Wang. Green Ferrate(VI) for Multiple Treatments of Fracturing Wastewater: Demulsification, Visbreaking, and Chemical Oxygen Demand Removal. *Int. J. Mol. Sci.* **2019**, *20*, 1857; https://doi.org/10.3390/ijms20081857 (Ref. **253** in the References list to the given article)

15. Arthur Merkel and Amir M. Ashrafi. An Investigation on the Application of Pulsed Electrodialysis Reversal in Whey Desalination. *Int. J. Mol. Sci.* **2019**, *20*, 1918; https://doi.org/10.3390/ijms20081918 (Ref. **62** in the References list to the given article)

16. Loïc Henaux, Jacinthe Thibodeau, Geneviève Pilon, Tom Gill, André Marette and Laurent Bazinet. How Charge and Triple Size-Selective Membrane Separation of Peptides from Salmon Protein Hydrolysate Orientate their Biological Response on Glucose Uptake. *Int. J. Mol. Sci.* **2019**, *20*, 1939; https://doi.org/10.3390/ijms20081939 (Ref. **63** in the References list to the given article)

17. Hong Zhang, Hong Liu, Chengshuo Shen, Fuwei Gan, Xuelei Su, Huibin Qiu, Bo Yang and Ping Yu. Chiral Recognition of Hexahelicene on a Surface via the Forming of Asymmetric Heterochiral Trimers. *Int. J. Mol. Sci.* **2019**, *20*, 2018; https://doi.org/10.3390/ijms20082018 (Ref. **2** in the References list to the given article)

18. Hongzhen Bai, Jianwei Wang, Zhongbao Li and Guping Tang. Macrocyclic Compounds for Drug and Gene Delivery in Immune-Modulating Therapy. *Int. J. Mol. Sci.* **2019**, *20*, 2097; https://doi.org/10.3390/ijms20092097 (Ref. **106** in the References list to the given article)

19. Shouyin Cai, Qizhong Tang, Sen Tian, Yiyu Lu and Xuechao Gao. Molecular Simulation Study on the Microscopic Structure and Mechanical Property of Defect-Containing sI Methane Hydrate. *Int. J. Mol. Sci.* **2019**, *20*, 2305; https://doi.org/10.3390/ijms20092305 (Ref. **107** in the References list to the given article)

20. Jongho Jeon. Review of Therapeutic Applications of Radiolabeled Functional Nanomaterials. *Int. J. Mol. Sci.* **2019**, *20*, 2323; https://doi.org/10.3390/ijms20092323 (Ref. **254** in the References list to the given article)

21. Pierre Magnico. Electro-Kinetic Instability in a Laminar Boundary Layer Next to an Ion Exchange Membrane. *Int. J. Mol. Sci.* **2019**, *20*, 2393; https://doi.org/10.3390/ijms20102393 (Ref. **26** in the References list to the given article)

22. Lorenzo Angiolini, Boiko Cohen and Abderrazzak Douhal. Single Crystal FLIM Characterization of Clofazimine Loaded in Silica-Based Mesoporous Materials and Zeolites. *Int. J. Mol. Sci.* **2019**, *20*, 2859; https://doi.org/10.3390/ijms20122859 (Ref. **108** in the References list to the given article)

23. Caixia Dong, Limin Han, Jucai Yang and Lin Cheng. Study on Structural Evolution, Thermochemistry and Electron Affinity of Neutral, Mono- and Di-Anionic Zirconium-Doped Silicon Clusters $ZrSi_n^{0/-/2-}$ (n = 6–16). *Int. J. Mol. Sci.* **2019**, *20*, 2933; https://doi.org/10.3390/ijms20122933 (Ref. **109** in the References list to the given article)

24. Yuto Kuwasaki, Keita Miyake, Keiji Fushimi, Yuka Takeda, Yoshibumi Ueda, Takahiro Nakajima, Masahiko Ikeuchi, Moritoshi Sato and Rei Narikawa. Protein Engineering of Dual-Cys Cyanobacteriochrome AM1_1186g2 for Biliverdin Incorporation and Far-Red/Blue Reversible Photoconversion. *Int. J. Mol. Sci.* **2019**, *20*, 2935; https://doi.org/10.3390/ijms20122935 (Ref. **255** in the References list to the given article)

25. Muhammad Ali Inam, Rizwan Khan, Muhammad Akram, Sarfaraz Khan and Ick Tae Yeom. Effect of Water Chemistry on Antimony Removal by Chemical Coagulation: Implications of ζ-Potential and Size of Precipitates. *Int. J. Mol. Sci.* **2019**, *20*, 2945; https://doi.org/10.3390/ijms20122945 (Ref. **27** in the References list to the given article)

26. Xin Liu, Cai Liu and Changgong Meng. Oligomerization of Silicic Acids in Neutral Aqueous Solution: A First-Principles Investigation. Xin Liu, Cai Liu and Changgong Meng. *Int. J. Mol. Sci.* **2019**, *20*, 3037; https://doi.org/10.3390/ijms20123037 (Ref. **110** in the References list to the given article)

27. Quan-De Wang, Yanjin Sun, Mao-Mao Sun and Jin-Hu Liang. Chemical Kinetics of Hydrogen Atom Abstraction from Propargyl Sites by Hydrogen and Hydroxy Radicals. *Int. J. Mol. Sci.* **2019**, *20*, 3227; https://doi.org/10.3390/ijms20133227 (Ref. **28** in the References list to the given article)

28. Wu-jhao Tien, Kun-you Chen, Fong-yin Huang and Chi-cheng Chiu. Effects of Cholesterol on Water Permittivity of Biomimetic Ion Pair Amphiphile Bilayers: Interplay between Membrane Bending and Molecular Packing. *Int. J. Mol. Sci.* **2019**, *20*, 3252; https://doi.org/10.3390/ijms20133252 (Ref. **64** in the References list to the given article)

29. Kai Yue, Xiaochen Sun, Jue Tang, Yiang Wei and Xinxin Zhang. A Simulation Study on the Interaction Between Pollutant Nanoparticles and the Pulmonary Surfactant Monolayer. *Int. J. Mol. Sci.* **2019**, *20*, 3281; https://doi.org/10.3390/ijms20133281 (Ref. **65** in the References list to the given article)

30. Babak Jaleh, Ehsan Sabzi Etivand, Bahareh Feizi Mohazzab, Mahmoud Nasrollahzadeh and Rajender S. Varma. Improving Wettability: Deposition of TiO2 Nanoparticles on the O2 Plasma Activated Polypropylene Membrane. *Int. J. Mol. Sci.* **2019**, *20*, 3309; https://doi.org/10.3390/ijms20133309 (Ref. **66** in the References list to the given article)

31. Shujahadeen B. Aziz, Muhamad H. Hamsan, Mohd F. Z. Kadir, Wrya O. Karim and Ranjdar M. Abdullah. Development of Polymer Blend Electrolyte Membranes Based on Chitosan: Dextran with High Ion Transport Properties for EDLC Application. *Int. J. Mol. Sci.* **2019**, *20*, 3369; https://doi.org/10.3390/ijms20133369 (Ref. **67** in the References list to the given article)

32. Jianguo Li, Hongying Dong, Fan Yang, Liangcheng Sun, Zhigang Zhao, Ruixi Bai and Hao Zhang. Simple Preparation of LaPO4:Ce, Tb Phosphors by an Ionic-Liquid-Driven Supported Liquid Membrane System. *Int. J. Mol. Sci.* **2019**, *20*, 3424; https://doi.org/10.3390/ijms20143424 (Ref. **68** in the References list to the given article)

33. Bartomeu Galmés, Antonio Franconetti and Antonio Frontera. Nitropyridine-1-Oxides as Excellent π-Hole Donors: Interplay between σ-Hole (Halogen, Hydrogen, Triel, and Coordination Bonds) and π-Hole Interactions. *Int. J. Mol. Sci.* **2019**, *20*, 3440; https://doi.org/10.3390/ijms20143440 (Ref. **191** in the References list to the given article)

34. Qianqian Ge, Xiang Zhu and Zhengjin Yang. Highly Conductive and Water-Swelling Resistant Anion Exchange Membrane for Alkaline Fuel Cells. *Int. J. Mol. Sci.* **2019**, *20*, 3470; https://doi.org/10.3390/ijms20143470 (Ref. **49** in the References list to the given article)

35. Lucie Vobecká, Tomáš Belloň and Zdeněk Slouka. Behavior of Embedded Cation-Exchange Particles in a DC Electric Field. *Int. J. Mol. Sci.* **2019**, *20*, 3579; https://doi.org/10.3390/ijms20143579 (Ref. **69** in the References list to the given article)

36. Olesya Rybalkina, Kseniya Tsygurina, Ekaterina Melnikova, Semyon Mareev, Ilya Moroz, Victor Nikonenko and Natalia Pismenskaya. Partial Fluxes of Phosphoric Acid Anions through Anion-Exchange Membranes in the Course of NaH_2PO_4 Solution Electrodialysis. *Int. J. Mol. Sci.* **2019**, *20*, 3593; https://doi.org/10.3390/ijms20143593 (Ref. **70** in the References list to the given article)

37. Ali Abbasi, Soraya Hosseini, Anongnat Somwangthanaroj, Ahmad Azmin Mohamad and Soorathep Kheawhom. Poly(2,6-Dimethyl-1,4-Phenylene Oxide)-Based Hydroxide Exchange Separator Membranes for Zinc–Air Battery. *Int. J. Mol. Sci.* **2019**, *20*, 3678; https://doi.org/10.3390/ijms20153678 (Ref. **50** in the References list to the given article)

38. Mimimorena Seggio, Antonia Nostro, Giovanna Ginestra, Fabiana Quaglia and Salvatore Sortino. Contact Lenses Delivering Nitric Oxide under Daylight for Reduction of Bacterial Contamination. *Int. J. Mol. Sci.* **2019**, *20*, 3735; https://doi.org/10.3390/ijms20153735 (Ref. **256** in the References list to the given article)

39. Zehua Wang, Chenxi Zhang, Guochun Lv, Xiaomin Sun, Ning Wang and Zhiqiang Li. Synergistic Reaction of SO_2 with NO_2 in Presence of H_2O and NH_3: A Potential Source of Sulfate Aerosol. *Int. J. Mol. Sci.* **2019**, *20*, 3746; https://doi.org/10.3390/ijms20153746 (Ref. **224** in the References list to the given article)

40. Krzysztof Żamojć, Magdalena Zdrowowicz, Aleksandra Hać, Maciej Witwicki, Paweł Błażej Rudnicki-Velasquez, Dariusz Wyrzykowski, Wiesław Wiczk and Lech Chmurzyński. Dihydroxy-Substituted Coumarins as Fluorescent Probes for Nanomolar-Level Detection of the 4-Amino-TEMPO Spin Label. *Int. J. Mol. Sci.* **2019**, *20*, 3802; https://doi.org/10.3390/ijms20153802 (Ref. **225** in the References list to the given article)

41. Philippe M. Heynderickx. Activity Coefficients for Liquid Organic Reactions: Towards a Better Understanding of True Kinetics with the Synthesis of Jasmin Aldehyde as Showcase. *Int. J. Mol. Sci.* **2019**, *20*, 3819; https://doi.org/10.3390/ijms20153819 (Ref. **29** in the References list to the given article)

42. M. A. Brza, Shujahadeen B. Aziz, H. Anuar and Muataz Hazza F. Al Hazza. From Green Remediation to Polymer Hybrid Fabrication with Improved Optical Band Gaps. *Int. J. Mol. Sci.* **2019**, *20*, 3910; https://doi.org/10.3390/ijms20163910 (Ref. **111** in the References list to the given article)

43. Bao-Ying Wang, Na Zhang, Zhen-Yu Li, Qiao-Lin Lang, Bing-Hua Yan, Yang Liu and Yang Zhang. Selective Separation of Acetic and Hexanoic Acids across Polymer Inclusion Membrane with Ionic Liquids as Carrier. *Int. J. Mol. Sci.* **2019**, *20*, 3915; https://doi.org/10.3390/ijms20163915 (Ref. **71** in the References list to the given article)

44. Antal Martinecz, Fabrizio Clarelli, Sören Abel and Pia Abel zur Wiesch. Reaction Kinetic Models of Antibiotic Heteroresistance. *Int. J. Mol. Sci.* **2019**, *20*, 3965; https://doi.org/10.3390/ijms20163965 (Ref. **30** in the References list to the given article)

45. Kyoung-Ho Park, Chan Joo Rhu, Jin Burm Kyong and Dennis N. Kevill. The Effect of the ortho Nitro Group in the Solvolysis of Benzyl and Benzoyl Halides. *Int. J. Mol. Sci.* **2019**, *20*, 4026; https://doi.org/10.3390/ijms20164026 (Ref. **31** in the References list to the given article)

46. Alfons Penzkofer, Arita Silapetere and Peter Hegemann. Absorption and Emission Spectroscopic Investigation of the Thermal Dynamics of the Archaerhodopsin 3 Based Fluorescent Voltage Sensor QuasAr1. Alfons Penzkofer, Arita Silapetere and Peter Hegemann. *Int. J. Mol. Sci.* **2019**, *20*, 4086; https://doi.org/10.3390/ijms20174086 (Ref. **112** in the References list to the given article)

47. Giulia Festa, Francesco Mallamace, Giulia Maria Sancesario, Carmelo Corsaro, Domenico Mallamace, Enza Fazio, Laura Arcidiacono, Victoria Garcia Sakai, Roberto Senesi, Enrico Preziosi, Giuseppe Sancesario and Carla Andreani. Aggregation States of Aβ1–40, Aβ1–42 and Aβp3–42 Amyloid Beta Peptides: A SANS Study. *Int. J. Mol. Sci.* **2019**, *20*, 4126; https://doi.org/10.3390/ijms20174126 (Ref. **113** in the References list to the given article)

48. Erika Reisz, Agnes Tekle-Röttering, Sergej Naumov, Winfried Schmidt and Torsten C. Schmidt. Reaction of 1-propanol with Ozone in Aqueous Media. *Int. J. Mol. Sci.* **2019**, *20*, 4165; https://doi.org/10.3390/ijms20174165 (Ref. **32** in the References list to the given article)

49. Yan Li, Zane Lombardo, Meera Joshi, Manju M. Hingorani and Ishita Mukerji. Mismatch Recognition by Saccharomyces cerevisiae Msh2-Msh6: Role of Structure and Dynamics. *Int. J. Mol. Sci.* **2019**, *20*, 4271; https://doi.org/10.3390/ijms20174271 (Ref. **237** in the References list to the given article)

50. Dylan Peukert, Ivan Kempson, Michael Douglass and Eva Bezak. Gold Nanoparticle Enhanced Proton Therapy: Monte Carlo Modeling of Reactive Species' Distributions Around a Gold Nanoparticle and the Effects of Nanoparticle Proximity and Clustering. *Int. J. Mol. Sci.* **2019**, *20*, 4280; https://doi.org/10.3390/ijms20174280 (Ref. **114** in the References list to the given article)

51. Yury A. Trofimov, Nikolay A. Krylov and Roman G. Efremov. Confined Dynamics of Water in Transmembrane Pore of TRPV1 Ion Channel. *Int. J. Mol. Sci.* **2019**, *20*, 4285; https://doi.org/10.3390/ijms20174285 (Ref. **72** in the References list to the given article)

52. Elham Shahhoseini, Bryce N. Feltis, Masao Nakayama, Terrence J. Piva, Dodie Pouniotis, Salem S. Alghamdi and Moshi Geso. Combined Effects of Gold Nanoparticles and Ionizing Radiation on Human Prostate and Lung Cancer Cell Migration. *Int. J. Mol. Sci.* **2019**, *20*, 4488; https://doi.org/10.3390/ijms20184488 (Ref. **115** in the References list to the given article)

53. Xiaoxiao Chen, Yanhui Sun, Youxiao Qi, Lin Liu, Fei Xu and Yan Zhao. Mechanistic and Kinetic Investigations on the Ozonolysis of Biomass Burning Products: Guaiacol, Syringol and Creosol. *Int. J. Mol. Sci.* **2019**, *20*, 4492; https://doi.org/10.3390/ijms20184492 (Ref. **33** in the References list to the given article)

54. Gloria Jiménez Sánchez, Pauline Maury, Lenka Stefancikova, Océane Campion, Gautier Laurent, Alicia Chateau, Farhan Bouraleh Hoch, Frédéric Boschetti, Franck Denat, Sophie Pinel, Jérôme Devy, Erika Porcel, Sandrine Lacombe, Rana Bazzi and Stéphane Roux. Fluorescent Radiosensitizing Gold Nanoparticles. *Int. J. Mol. Sci.* **2019**, *20*, 4618; https://doi.org/10.3390/ijms20184618 (Ref. **116** in the References list to the given article)

55. Giuseppina Raffaini and Fabio Ganazzoli. A Molecular Dynamics Study of a Photodynamic Sensitizer for Cancer Cells: Inclusion Complexes of γ-Cyclodextrins with C70. *Int. J. Mol. Sci.* **2019**, *20*, 4831; https://doi.org/10.3390/ijms20194831 (Ref. **257** in the References list to the given article)

56. Agnieszka Czapik, Maciej Jelecki and Marcin Kwit. Chiral Cocrystal Solid Solutions, Molecular Complexes, and Salts of N-Triphenylacetyl-l-Tyrosine and Diamines. *Int. J. Mol. Sci.* **2019**, *20*, 5004; https://doi.org/10.3390/ijms20205004 (Ref. **3** in the References list to the given article)

57. Jyung-Hurng Liu, Jun-Yi Yang, Duen-Wei Hsu, Yi-Hua Lai, Yun-Pei Li, Yi-Rung Tsai and Ming-Hon Hou. Crystal Structure-Based Exploration of Arginine-Containing Peptide Binding in the ADP-Ribosyltransferase Domain of the Type III Effector XopAI Protein. *Int. J. Mol. Sci.* **2019**, *20*, 5085; https://doi.org/10.3390/ijms20205085 (Ref. **258** in the References list to the given article)

58. Pascual García-Pérez, Sonia Losada-Barreiro, Pedro P. Gallego and Carlos Bravo-Díaz. Cyclodextrin-Elicited Bryophyllum Suspension Cultured Cells: Enhancement of the Production of Bioactive Compounds. *Int. J. Mol. Sci.* **2019**, *20*, 5180; https://doi.org/10.3390/ijms20205180 (Ref. **259** in the References list to the given article)

59. Akikazu Sakudo, Yoshihito Yagyu and Takashi Onodera. Disinfection and Sterilization Using Plasma Technology: Fundamentals and Future Perspectives for Biological Applications. *Int. J. Mol. Sci.* **2019**, *20*, 5216; https://doi.org/10.3390/ijms20205216 (Ref. **238** in the References list to the given article)

60. Mark Tingey, Krishna C. Mudumbi, Eric C. Schirmer and Weidong Yang. Casting a Wider Net: Differentiating between Inner Nuclear Envelope and Outer Nuclear Envelope Transmembrane Proteins. *Int. J. Mol. Sci.* **2019**, *20*, 5248; https://doi.org/10.3390/ijms20215248 (Ref. **72** in the References list to the given article)

61. Shujahadeen B. Aziz, Wrya O. Karim, M.A. Brza, Rebar T. Abdulwahid, Salah Raza Saeed, Shakhawan Al-Zangana and M.F.Z. Kadir. Ion Transport Study in CS: POZ Based Polymer Membrane Electrolytes Using Trukhan Model. *Int. J. Mol. Sci.* **2019**, *20*, 5265; https://doi.org/10.3390/ijms20215265 (Ref. **60** in the References list to the given article)

62. Sow-Hsin Chen, Carmelo Corsaro, Francesco Mallamace, Enza Fazio and Domenico Mallamace. The Proton Density of States in Confined Water (H_2O). *Int. J. Mol. Sci.* **2019**, *20*, 5373; https://doi.org/10.3390/ijms20215373 (Ref. **239** in the References list to the given article)

63. Manuel Vicente, Jussep Salgado-Almario, Joaquim Soriano, Miguel Burgos, Beatriz Domingo and Juan Llopis. Visualization of Mitochondrial Ca2+ Signals in Skeletal Muscle of Zebrafish Embryos with Bioluminescent Indicators. *Int. J. Mol. Sci.* **2019**, *20*, 5409; https://doi.org/10.3390/ijms20215409 (Ref. **260** in the References list to the given article)

64. Qingqing Chen, Yan Wang, Shanshan Shi, Kaihang Li, Ling Zhang and Jian Gao. Insights into the Interaction Mechanisms of the Proviral Integration Site of Moloney Murine Leukemia Virus (Pim) Kinases with Pan-Pim Inhibitors PIM447 and AZD1208: A Molecular Dynamics Simulation and MM/GBSA Calculation Study. *Int. J. Mol. Sci.* **2019**, *20*, 5410; https://doi.org/10.3390/ijms20215410 (Ref. **117** in the References list to the given article)

65. Lili Du, Zhiping Yan, Xueqin Bai, Runhui Liang and David Lee Phillips. Time-Resolved Spectroscopic Study of N,N–Di(4–bromo)nitrenium Ions in Acidic Aqueous Solution. *Int. J. Mol. Sci.* **2019**, *20*, 5512; https://doi.org/10.3390/ijms20215512 (Ref. **118** in the References list to the given article)

66. Tünde Szatmári, Rita Hargitai, Géza Sáfrány and Katalin Lumniczky. Extracellular Vesicles in Modifying the Effects of Ionizing Radiation. *Int. J. Mol. Sci.* **2019**, *20*, 5527; https://doi.org/10.3390/ijms20225527 (Ref. **261** in the References list to the given article)

67. Pin Shao and Lepeng Jiang. Flow and Mixing Behavior in a New Bottom Blown Copper Smelting Furnace. *Int. J. Mol. Sci.* **2019**, *20*, 5757; https://doi.org/10.3390/ijms20225757 (Ref. **34** in the References list to the given article)

68. Samhitha Kancharla, Emmanuel Canales and Paschalis Alexandridis. Perfluorooctanoate in Aqueous Urea Solutions: Micelle Formation, Structure, and Microenvironment. *Int. J. Mol. Sci.* **2019**, *20*, 5761; https://doi.org/10.3390/ijms20225761 (Ref. **4** in the References list to the given article)

69. Fabao Luo, Yang Wang, Maolin Sha and Yanxin Wei. Correlations of Ion Composition and Power Efficiency in a Reverse Electrodialysis Heat Engine. *Int. J. Mol. Sci.* **2019**, *20*, 5860; https://doi.org/10.3390/ijms20235860 (Ref. **75** in the References list to the given article)

70. Ana Maria Toader, Maria Cristina Buta, Dan Maftei, Mihai V. Putz and Fanica Cimpoesu. Atoms in Generalized Orbital Configurations: Towards Atom-Dedicated Density Functionals. *Int. J. Mol. Sci.* **2019**, *20*, 5943; https://doi.org/10.3390/ijms20235943 (Ref. **192** in the References list to the given article)

71. Suraj Kannath, Paweł Adamczyk, Langping Wu, Hans H. Richnow and Agnieszka Dybala-Defratyka. Can Alkaline Hydrolysis of γ-HCH Serve as a Model Reaction to Study Its Aerobic Enzymatic

Dehydrochlorination by LinA? *Int. J. Mol. Sci.* **2019**, *20*, 5955; https://doi.org/10.3390/ijms20235955 (Ref. **35** in the References list to the given article)

72. Elaine Fabre, Carlos Vale, Eduarda Pereira and Carlos M. Silva. Experimental Measurement and Modeling of Hg(II) Removal from Aqueous Solutions Using Eucalyptus globulus Bark: Effect of pH, Salinity and Biosorbent Dosage. *Int. J. Mol. Sci.* **2019**, *20*, 5973; https://doi.org/10.3390/ijms20235973 (Ref. **36** in the References list to the given article)
73. Zhen-Nan Tian, Ding-Qi Wu, Xue-Jiao Sun, Ting-Ting Liu and Zhi-Yong Xing. A Benzothiazole-Based Fluorescent Probe for Ratiometric Detection of Al^{3+} and Its Application in Water Samples and Cell Imaging. *Int. J. Mol. Sci.* **2019**, *20*, 5993; https://doi.org/10.3390/ijms20235993 (Ref. **240** in the References list to the given article)
74. Xuesong Cao, Chenxi Zhang, Zehua Wang and Xiaomin Sun. Catalytic Reaction Mechanism of NO–CO on the ZrO_2 (110) and (111) Surfaces. *Int. J. Mol. Sci.* **2019**, *20*, 6129; https://doi.org/10.3390/ijms20246129 (Ref. **193** in the References list to the given article)
75. Kristina Vlahoviček-Kahlina, Josipa Suć Sajko and Ivanka Jerić. C-Linked Glycomimetic Libraries Accessed by the Passerini Reaction. *Int. J. Mol. Sci.* **2019**, *20*, 6236; https://doi.org/10.3390/ijms20246236 (Ref. **119** in the References list to the given article)
76. Aleksandra M. Bondžić, Andreja R. Leskovac, Sandra Ž. Petrović, Dragana D. Vasić Anićijević, Marco Luce, Lara Massai, Amanda Generosi, Barbara Paci, Antonio Cricenti, Luigi Messori and Vesna M. Vasić. Conjugates of Gold Nanoparticles and Antitumor Gold(III) Complexes as a Tool for Their AFM and SERS Detection in Biological Tissue. *Int. J. Mol. Sci.* **2019**, *20*, 6306; https://doi.org/10.3390/ijms20246306 (Ref. **120** in the References list to the given article)
77. B. Roy Frieden and Robert Gatenby. Ion-Based Cellular Signal Transmission, Principles of Minimum Information Loss, and Evolution by Natural Selection. *Int. J. Mol. Sci.* **2020**, *21*, 9; https://doi.org/10.3390/ijms21010009 (Ref. **76** in the References list to the given article)
78. Alfons Penzkofer, Arita Silapetere and Peter Hegemann. Photocycle Dynamics of the Archaerhodopsin 3 Based Fluorescent Voltage Sensor QuasAr1. *Int. J. Mol. Sci.* **2020**, *21*, 160; https://doi.org/10.3390/ijms21010160 (Ref. **121** in the References list to the given article)
79. Francis Boateng and Wilfred Ngwa. Delivery of Nanoparticle-Based Radiosensitizers for Radiotherapy Applications. *Int. J. Mol. Sci.* **2020**, *21*, 273; https://doi.org/10.3390/ijms21010273 (Ref. **122** in the References list to the given article)
80. Carlos Diaz-Uribe, William Vallejo and Cesar Quiñones. Physical-Chemical Study of Anthracene Selective Oxidation by a Fe(III)-Phenylporhyrin Derivative. *Int. J. Mol. Sci.* **2020**, *21*, 353; https://doi.org/10.3390/ijms21010353 (Ref. **37** in the References list to the given article)
81. Praveen Kumar Nattramilarasu, Roland Bücker, Fábia Daniela Lobo de Sá, Anja Fromm, Oliver Nagel, In-Fah Maria Lee, Eduard Butkevych, Soraya Mousavi, Claudia Genger, Sigri Kløve, Markus M. Heimesaat, Stefan Bereswill, Michal R. Schweiger, Hans Linde Nielsen, Hanno Troeger and Jörg-Dieter Schulzke. Campylobacter concisus Impairs Sodium Absorption in Colonic Epithelium via ENaC Dysfunction and Claudin-8 Disruption. *Int. J. Mol. Sci.* **2020**, *21*, 373; https://doi.org/10.3390/ijms21020373 (Ref. **77** in the References list to the given article)
82. Archana Verma, John P. Stoppelman and Jesse G. McDaniel. Tuning Water Networks via Ionic Liquid/Water Mixtures. *Int. J. Mol. Sci.* **2020**, *21*, 403; https://doi.org/10.3390/ijms21020403 (Ref. **123** in the References list to the given article)
83. Farasat Kazmi, Katherine A. Vallis, Balamurugan A. Vellayappan, Aishwarya Bandla, Duan Yukun and Robert Carlisle. Megavoltage Radiosensitization of Gold Nanoparticles on a Glioblastoma Cancer Cell Line Using a Clinical Platform. *Int. J. Mol. Sci.* **2020**, *21*, 429; https://doi.org/10.3390/ijms21020429 (Ref. **124** in the References list to the given article)
84. Marina V. Maslova, Vladimir I. Ivanenko, Nataliya Yu. Yanicheva and Natalia V. Mudruk. Comparison of The Sorption Kinetics of Lead(II) and Zinc(II) on Titanium Phosphate Ion-Exchanger. Marina V. Maslova, Vladimir I. Ivanenko, Nataliya Yu. Yanicheva and Natalia V. Mudruk. *Int. J. Mol. Sci.* **2020**, *21*, 447; https://doi.org/10.3390/ijms21020447 (Ref. **38** in the References list to the given article)

85. Sokratis T. Tsantis, Demetrios I. Tzimopoulos, Malgorzata Holynska and Spyros P. Perlepes. Oligonuclear Actinoid Complexes with Schiff Bases as Ligands—Older Achievements and Recent Progress. *Int. J. Mol. Sci.* **2020**, *21*, 555; https://doi.org/10.3390/ijms21020555 (Ref. **125** in the References list to the given article)

86. Douglas Howard, Sonia Sebastian, Quy Van-Chanh Le, Benjamin Thierry and Ivan Kempson. Chemical Mechanisms of Nanoparticle Radiosensitization and Radioprotection: A Review of Structure-Function Relationships Influencing Reactive Oxygen Species. *Int. J. Mol. Sci.* **2020**, *21*, 579; https://doi.org/10.3390/ijms21020579 (Ref. **126** in the References list to the given article)

87. Francesco Mallamace, Carmelo Corsaro, Domenico Mallamace, Enza Fazio, Sow-Hsin Chen and Antonio Cupane. Specific Heat and Transport Functions of Water. *Int. J. Mol. Sci.* **2020**, *21*, 622; https://doi.org/10.3390/ijms21020622 (Ref. **5** in the References list to the given article)

88. Erik Antonio Vázquez-Montelongo, José Enrique Vázquez-Cervantes and G. Andrés Cisneros. Current Status of AMOEBA–IL: A Multipolar/Polarizable Force Field for Ionic Liquids. *Int. J. Mol. Sci.* **2020**, *21*, 697; https://doi.org/10.3390/ijms21030697 (Ref. **194** in the References list to the given article)

89. Dongxu Han, Zhongbao Han, Liyan Liu, Ying Wang, Shigang Xin, Hongbo Zhang and Zhan Yu. Solubility Enhancement of Myricetin by Inclusion Complexation with Heptakis-O-(2-Hydroxypropyl)-β-Cyclodextrin: A Joint Experimental and Theoretical Study. *Int. J. Mol. Sci.* **2020**, *21*, 766; https://doi.org/10.3390/ijms21030766 (Ref. **262** in the References list to the given article)

90. Isabel Iglesias, José A. Huidobro, Belén F. Alfonso, Camino Trobajo, Aránzazu Espina, Rafael Mendoza and José R. García. Kinetic Analysis of the Thermal Decomposition of Iron(III) Phosphates: $Fe(NH_3)_2PO_4$ and $Fe(ND_3)_2PO_4$. *Int. J. Mol. Sci.* **2020**, *21*, 781; https://doi.org/10.3390/ijms21030781 (Ref. **39** in the References list to the given article)

91. Zhidong Zhang and Ueli Angst. Modeling Anomalous Moisture Transport in Cement-Based Materials with Kinetic Permeability. *Int. J. Mol. Sci.* **2020**, *21*, 837; https://doi.org/10.3390/ijms21030837 (Ref. **40** in the References list to the given article)

92. Yuika Onami, Takayasu Kawasaki, Hiroki Aizawa, Tomoyuki Haraguchi, Takashiro Akitsu, Koichi Tsukiyama and Mauricio A. Palafox. Degradation of Human Serum Albumin by Infrared Free Electron Laser Enhanced by Inclusion of a Salen-Type Schiff Base Zn(II) Complex. *Int. J. Mol. Sci.* **2020**, *21*, 874; https://doi.org/10.3390/ijms21030874 (Ref. **195** in the References list to the given article)

93. Thalita Sévia Soares de Almeida Magalhães, Pollyana Cristina de Oliveira Macedo, Stephany Yumi Kawashima Pacheco, Sofia Santos da Silva, Euzébio Guimarães Barbosa, Rayanne Rocha Pereira, Roseane Maria Ribeiro Costa, José Otávio Carréra Silva Junior, Marília Andreza da Silva Ferreira, José Cezário de Almeida, Pedro José Rolim Neto, Attilio Converti and Ádley Antonini Neves de Lima. Development and Evaluation of Antimicrobial and Modulatory Activity of Inclusion Complex of Euterpe oleracea Mart Oil and β-Cyclodextrin or HP-β-Cyclodextrin. *Int. J. Mol. Sci.* **2020**, *21*, 942; https://doi.org/10.3390/ijms21030942 (Ref. **127** in the References list to the given article)

94. Svetlana Zyryanova, Semyon Mareev, Violetta Gil, Elizaveta Korzhova, Natalia Pismenskaya, Veronika Sarapulova, Olesya Rybalkina, Evgeniy Boyko, Christian Larchet, Lasaad Dammak and Victor Nikonenko. How Electrical Heterogeneity Parameters of Ion-Exchange Membrane Surface Affect the Mass Transfer and Water Splitting Rate in Electrodialysis. Svetlana Zyryanova, Semyon Mareev, Violetta Gil, Elizaveta Korzhova, Natalia Pismenskaya, Veronika Sarapulova, Olesya Rybalkina, Evgeniy Boyko, Christian Larchet, Lasaad Dammak and Victor Nikonenko. *Int. J. Mol. Sci.* **2020**, *21*, 973; https://doi.org/10.3390/ijms21030973 (Ref. **78** in the References list to the given article)

95. Kaizheng Wang, Feipeng Wang, Ziyi Lou, Qiuhuang Han, Qi Zhao, Kelin Hu, Zhengyong Huang and Jian Li. Relationship between the Electrical Characteristics of Molecules and Fast Streamers in Ester Insulation Oil. *Int. J. Mol. Sci.* **2020**, *21*, 974; https://doi.org/10.3390/ijms21030974 (Ref. **196** in the References list to the given article)

96. Changsun Eun. Effects of the Size, the Number, and the Spatial Arrangement of Reactive Patches on a Sphere on Diffusion-Limited Reaction Kinetics: A Comprehensive Study. *Int. J. Mol. Sci.* **2020**, *21*, 997; https://doi.org/10.3390/ijms21030997 (Ref. **41** in the References list to the given article)

97. Lilei Zhang, Jingxiao Zhang, Yuanyu Xia, Menghan Xun, Hong Chen, Xianghui Liu and Xia Yin. Metal-Free Carbon Quantum Dots Implant Graphitic Carbon Nitride: Enhanced Photocatalytic Dye Wastewater

Purification with Simultaneous Hydrogen Production. *Int. J. Mol. Sci.* **2020**, *21*, 1052; https://doi.org/10.3390/ijms21031052 (Ref. **241** in the References list to the given article)

98. Caroline Velez, Brian Doherty and Orlando Acevedo. Accurate Diels-Alder Energies and Endo Selectivity in Ionic Liquids Using the OPLS-VSIL Force Field. *Int. J. Mol. Sci.* **2020**, *21*, 1190; https://doi.org/10.3390/ijms21041190 (Ref. **197** in the References list to the given article)

99. Concetta Imperatore,Mohammadhassan Valadan, Luciana Tartaglione, Marco Persico, Anna Ramunno, Marialuisa Menna, Marcello Casertano, Carmela Dell'Aversano, Manjot Singh, Maria Luisa d'Aulisio Garigliota, Francesco Bajardi, Elena Morelli, Caterina Fattorusso, Carlo Altucci and Michela Varra. Exploring the Photodynamic Properties of Two Antiproliferative Benzodiazopyrrole Derivatives. *Int. J. Mol. Sci.* **2020**, *21*, 1246; https://doi.org/10.3390/ijms21041246 (Ref. **128** in the References list to the given article)

100. Mohamed E. Elshakre, Mahmoud A. Noamaan, Hussein Moustafa and Haider Butt. Density Functional Theory, Chemical Reactivity, Pharmacological Potential and Molecular Docking of Dihydrothiouracil-Indenopyridopyrimidines with Human-DNA Topoisomerase II. *Int. J. Mol. Sci.* **2020**, *21*, 1253; https://doi.org/10.3390/ijms21041253 (Ref. **198** in the References list to the given article)

101. Oleg V. Mikhailov and Denis V. Chachkov. M(VI) Oxidation State Stabilization in Iron, Cobalt and Nickel Heteroligand Metal Chelates Containing 3,7,11,15-Tetraazaporphine and Two Axial Oxo Ligands: Quantum-Chemical Simulation. *Int. J. Mol. Sci.* **2020**, *21*, 1494; https://doi.org/10.3390/ijms21041494 (Ref. **129** in the References list to the given article)

102. Si-Ming Liao, Bo Lu, Xue-Hui Liu, Zhi-Long Lu, Shi-Jie Liang, Dong Chen, Frederic A. Troy II, Ri-Bo Huang and Guo-Ping Zhou. Molecular Interactions of the Polysialytransferase Domain (PSTD) in ST8Sia IV with CMP-Sialic Acid and Polysialic Acid Required for Polysialylation of the Neural Cell Adhesion Molecule Proteins: An NMR Study. *Int. J. Mol. Sci.* **2020**, *21*, 1590; https://doi.org/10.3390/ijms21051590 (Ref. **6** in the References list to the given article)

103. Amlan Chakraborty, Simon G. Royce, Cordelia Selomulya and Magdalena Plebanski. A Novel Approach for Non-Invasive Lung Imaging and Targeting Lung Immune Cells. *Int. J. Mol. Sci.* **2020**, *21*, 1613; https://doi.org/10.3390/ijms21051613 (Ref. **263** in the References list to the given article)

104. Xiaomin Yang, Daniela Salado-Leza, Erika Porcel, César R. González-Vargas, Farah Savina, Diana Dragoe, Hynd Remita and Sandrine Lacombe. A Facile One-Pot Synthesis of Versatile PEGylated Platinum Nanoflowers and Their Application in Radiation Therapy. *Int. J. Mol. Sci.* **2020**, *21*, 1619; https://doi.org/10.3390/ijms21051619 (Ref. **264** in the References list to the given article)

105. Elias Lüthi, Paola Andrea Forero Cortés, Alessandro Prescimone, Edwin C. Constable and Catherine E. Housecroft. Schiff Base Ancillary Ligands in Bis(diimine) Copper(I) Dye-Sensitized Solar Cells. *Int. J. Mol. Sci.* **2020**, *21*, 1735; https://doi.org/10.3390/ijms21051735 (Ref. **130** in the References list to the given article)

106. Krzysztof Szafrański, Jarosław Sławiński, Łukasz Tomorowicz and Anna Kawiak. Synthesis, Anticancer Evaluation and Structure-Activity Analysis of Novel (E)- 5-(2-Arylvinyl)-1,3,4-oxadiazol-2-yl)benzenesulfonamides. *Int. J. Mol. Sci.* **2020**, *21*, 2235; https://doi.org/10.3390/ijms21062235 (Ref. **132** in the References list to the given article)

107. Oleg V. Mikhailov. Preface from the Guest Editor of Special Issue "Quantum-Chemical Modeling and Design of Chelate and Macrocyclic Metal Complexes". *Int. J. Mol. Sci.* **2020**, *21*, 2339; https://doi.org/10.3390/ijms21072339 (Ref. **199** in the References list to the given article)

108. Takumi Ishida, Yuta Sawanaka, Ryota Toyama, Zhenfei Ji, Hiroki Mori and Yasushi Nishihara. Synthesis of Dinaphtho[2,3-d:2',3'-d']anthra[1,2-b:5,6-b']dithiophene (DNADT) Derivatives: Effect of Alkyl Chains on Transistor Properties. *Int. J. Mol. Sci.* **2020**, *21*, 2447; https://doi.org/10.3390/ijms21072447 (Ref. **132** in the References list to the given article)

109. Shuheng Huang, Linxin Chen, Hu Mei, Duo Zhang, Tingting Shi, Zuyin Kuang, Yu Heng, Lei Xu and Xianchao Pan. In Silico Prediction of the Dissociation Rate Constants of Small Chemical Ligands by 3D-Grid-Based VolSurf Method. *Int. J. Mol. Sci.* **2020**, *21*, 2456; https://doi.org/10.3390/ijms21072456 (Ref. **133** in the References list to the given article)

110. Jiří Czernek and Jiří Brus. Monitoring the Site-Specific Solid-State NMR Data in Oligopeptides. *Int. J. Mol. Sci.* **2020**, *21*, 2700; https://doi.org/10.3390/ijms21082700 (Ref. **134** in the References list to the given article)

111. Barbara Pucelik, Adam Sułek, Agnieszka Drozd, Grażyna Stochel, Mariette M. Pereira, Sara M.A. Pinto, Luis G. Arnaut and Janusz M. Dąbrowski. Enhanced Cellular Uptake and Photodynamic Effect with Amphiphilic Fluorinated Porphyrins: The Role of Sulfoester Groups and the Nature of Reactive Oxygen Species. *Int. J. Mol. Sci.* **2020**, *21*, 2786; https://doi.org/10.3390/ijms21082786 (Ref. **135** in the References list to the given article)

112. Claudie Aspirault, Alain Doyen and Laurent Bazinet. Impact of Preheating Temperature on the Separation of Whey Proteins When Combined with Chemical or Bipolar Membrane Electrochemical Acidification. *Int. J. Mol. Sci.* **2020**, *21*, 2792; https://doi.org/10.3390/ijms21082792 (Ref. **79** in the References list to the given article)

113. Keunhong Jeong, Hye Jin Jeong, Seung Min Woo and Sungchul Bae. Prediction of Binding Stability of Pu(IV) and PuO_2(VI) by Nitrogen Tridentate Ligands in Aqueous Solution. *Int. J. Mol. Sci.* **2020**, *21*, 2791; https://doi.org/10.3390/ijms21082791 (Ref. **136** in the References list to the given article)

114. Manas Sutradhar, Tannistha Roy Barman, Armando J.L. Pombeiro and Luísa M.D.R.S. Martins. Aroylhydrazone Schiff Base Derived Cu(II) and V(V) Complexes: Efficient Catalysts towards Neat Microwave-Assisted Oxidation of Alcohols. *Int. J. Mol. Sci.* **2020**, *21*, 2832; https://doi.org/10.3390/ijms21082832 (Ref. **137** in the References list to the given article)

115. Elisa Fresch and Elisabetta Collini. Relaxation Dynamics of Chlorophyll b in the Sub-ps Ultrafast Timescale Measured by 2D Electronic Spectroscopy. Elisa Fresch and Elisabetta Collini. *Int. J. Mol. Sci.* **2020**, *21*, 2836; https://doi.org/10.3390/ijms21082836 (Ref. **138** in the References list to the given article)

116. Veerappan Mani, T.S.T. Balamurugan and Sheng-Tung Huang. Rapid One-Pot Synthesis of Polydopamine Encapsulated Carbon Anchored with Au Nanoparticles: Versatile Electrocatalysts for Chloramphenicol and Folic Acid Sensors. *Int. J. Mol. Sci.* **2020**, *21*, 2853; https://doi.org/10.3390/ijms21082853 (Ref. **265** in the References list to the given article)

117. Nanoparticle-Based Radiosensitization. Ivan Kempson. *Int. J. Mol. Sci.* **2020**, *21*, 2879; https://doi.org/10.3390/ijms21082879 (Ref. **266** in the References list to the given article)

118. Arseniy A. Otlyotov, Igor V. Ryzhov, Ilya A. Kuzmin, Yuriy A. Zhabanov, Maxim S. Mikhailov and Pavel A. Stuzhin. DFT Study of Molecular and Electronic Structure of Ca(II) and Zn(II) Complexes with Porphyrazine and tetrakis(1,2,5-thiadiazole)porphyrazine. *Int. J. Mol. Sci.* **2020**, *21*, 2923; https://doi.org/10.3390/ijms21082923 (Ref. **200** in the References list to the given article)

119. Jean Michel Merkes, Leiming Zhu, Srishti Ballabh Bahukhandi, Magnus Rueping, Fabian Kiessling and Srinivas Banala. Photoacoustic Imaging Probes Based on Tetrapyrroles and Related Compounds. *Int. J. Mol. Sci.* **2020**, *21*, 3082; https://doi.org/10.3390/ijms21093082 (Ref. **139** in the References list to the given article)

120. Ryan Dula Corpuz, Lyn Marie De Juan-Corpuz, Mai Thanh Nguyen, Tetsu Yonezawa, Heng-Liang Wu, Anongnat Somwangthanaroj and Soorathep Kheawhom. Binder-Free α-MnO_2 Nanowires on Carbon Cloth as Cathode Material for Zinc-Ion Batteries. *Int. J. Mol. Sci.* **2020**, *21*, 3113; https://doi.org/10.3390/ijms21093113 (Ref. **267** in the References list to the given article)

121. Zineb Felfli, Kelvin Suggs, Nantambu Nicholas and Alfred Z. Msezane. Fullerene Negative Ions: Formation and Catalysis. *Int. J. Mol. Sci.* **2020**, *21*, 3159; https://doi.org/10.3390/ijms21093159 (Ref. **226** in the References list to the given article)

122. Eva Molnar, Emese Gal, Luiza Gaina, Castelia Cristea, Eva Fischer-Fodor, Maria Perde-Schrepler, Patriciu Achimas-Cadariu, Monica Focsan and Luminita Silaghi-Dumitrescu. Novel Phenothiazine-Bridged Porphyrin-(Hetero)aryl dyads: Synthesis, Optical Properties, In Vitro Cytotoxicity and Staining of Human Ovarian Tumor Cell Lines. *Int. J. Mol. Sci.* **2020**, *21*, 3178; https://doi.org/10.3390/ijms21093178 (Ref. **140** in the References list to the given article)

123. Tse-Wei Chen, Elayappan Tamilalagan, Shen-Ming Chen, Muthumariappan Akilarasan, Selvarasu Maheshwaran and Xiaoheng Liu. An Ultra-Sensitive Electrochemical Sensor for the Detection of Carcinogen Oxidative Stress 4-Nitroquinoline N-Oxide in Biologic Matrices Based on Hierarchical Spinel Structured $NiCo_2O_4$ and $NiCo_2S_4$; A Comparative Study. *Int. J. Mol. Sci.* **2020**, *21*, 3273; https://doi.org/10.3390/ijms21093273 (Ref. **51** in the References list to the given article)

124. Lei Wang, Liyun Zhang, Yuke Wang, Yahong Ou, Xu Wang, Yuanhu Pan, Yulian Wang, Lingli Huang, Guyue Cheng, Shuyu Xie, Dongmei Chen and Yanfei Tao. Construction of an Electrochemical Receptor Sensor Based on Graphene/Thionine for the Sensitive Determination of β-Lactam Antibiotics Content in Milk.

Int. J. Mol. Sci. **2020**, *21*, 3306; https://doi.org/10.3390/ijms21093306 (Ref. **52** in the References list to the given article)

125. Hanieh Montaseri, Cherie Ann Kruger and Heidi Abrahamse. Recent Advances in Porphyrin-Based Inorganic Nanoparticles for Cancer Treatment. *Int. J. Mol. Sci.* **2020**, *21*, 3358; https://doi.org/10.3390/ijms21093358 (Ref. **141** in the References list to the given article)

126. Barbara Miroslaw. Homo- and Hetero-Oligonuclear Complexes of Platinum Group Metals (PGM) Coordinated by Imine Schiff Base Ligands. *Int. J. Mol. Sci.* **2020**, *21*, 3493; https://doi.org/10.3390/ijms21103493 (Ref. **142** in the References list to the given article)

127. Bianca Patrascu, Sorin Mocanu, Anca Coman, Augustin M. Madalan, Codruta Popescu, Anca Paun, Mihaela Matache and Petre Ionita. Synthesis of Fluorescent Dansyl Derivatives of Methoxyamine and Diphenylhydrazine as Free Radical Precursors. *Int. J. Mol. Sci.* **2020**, *21*, 3559; https://doi.org/10.3390/ijms21103559 (Ref. **227** in the References list to the given article)

128. Samira Gholizadeh Dogaheh, Sara Barbero, Joel Barrientos, Jan Janczak, Janet Soleimannejad and E. Carolina Sañudo. Cathecol and Naphtol Groups in Salphen-Type Schiff Bases for the Preparation of Polynuclear Complexes. *Int. J. Mol. Sci.* **2020**, *21*, 3574; https://doi.org/10.3390/ijms21103574 (Ref. **143** in the References list to the given article)

129. Massimiliano Gaeta, Giuseppe Sanfilippo, Aurore Fraix, Giuseppe Sortino, Matteo Barcellona, Gea Oliveri Conti, Maria Elena Fragalà, Margherita Ferrante, Roberto Purrello and Alessandro D'Urso. Photodegradation of Antibiotics by Noncovalent Porphyrin-Functionalized TiO2 in Water for the Bacterial Antibiotic Resistance Risk Management. *Int. J. Mol. Sci.* **2020**, *21*, 3775; https://doi.org/10.3390/ijms21113775 (Ref. **268** in the References list to the given article)

130. Areej A. Eskandrani, Shimaa M. Ali and Hibah M. Al-Otaibi. Study of the Oxygen Evolution Reaction at Strontium Palladium Perovskite Electrocatalyst in Acidic Medium. *Int. J. Mol. Sci.* **2020**, *21*, 3785; https://doi.org/10.3390/ijms21113785 (Ref. **53** in the References list to the given article)

131. Brian Brennan, Ciprian Briciu-Burghina, Sean Hickey, Thomas Abadie, Sultan M. al Ma Awali, Yan Delaure, John Durkan, Linda Holland, Brid Quilty, Mohammad Tajparast, Casper Pulit, Lorna Fitzsimons, Kieran Nolan, Fiona Regan and Jenny Lawler. Pilot Scale Study: First Demonstration of Hydrophobic Membranes for the Removal of Ammonia Molecules from Rendering Condensate Wastewater. *Int. J. Mol. Sci.* **2020**, *21*, 3914; https://doi.org/10.3390/ijms21113914 (Ref. **80** in the References list to the given article)

132. Nile S. Abularrage, Brian J. Levandowski and Ronald T. Raines. Synthesis and Diels–Alder Reactivity of 4-Fluoro-4-Methyl-4H-Pyrazoles. *Int. J. Mol. Sci.* **2020**, *21*, 3964; https://doi.org/10.3390/ijms21113964 (Ref. **144** in the References list to the given article)

133. Ilaria Giuseppina Occhiuto, Maria Angela Castriciano, Mariachiara Trapani, Roberto Zagami, Andrea Romeo, Robert F. Pasternack and Luigi Monsù Scolaro. Controlling J-Aggregates Formation and Chirality Induction through Demetallation of a Zinc(II) Water Soluble Porphyrin. *Int. J. Mol. Sci.* **2020**, *21*, 4001; https://doi.org/10.3390/ijms21114001 (Ref. **145** in the References list to the given article)

134. Zaira Gadzhimagomedova, Peter Zolotukhin, Oleg Kit, Daria Kirsanova and Alexander Soldatov. Nanocomposites for X-Ray Photodynamic Therapy. *Int. J. Mol. Sci.* **2020**, *21*, 4004; https://doi.org/10.3390/ijms21114004 (Ref. **269** in the References list to the given article)

135. Lei Xie, Nan Xiao, Lu Li, Xinan Xie and Yan Li. Theoretical Insight into the Interaction between Chloramphenicol and Functional Monomer (Methacrylic Acid) in Molecularly Imprinted Polymers. *Int. J. Mol. Sci.* **2020**, *21*, 4139; https://doi.org/10.3390/ijms21114139 (Ref. **201** in the References list to the given article)

136. Jolanta Flieger, Joanna Feder-Kubis and Małgorzata Tatarczak-Michalewska. Chiral Ionic Liquids: Structural Diversity, Properties and Applications in Selected Separation Techniques. *Int. J. Mol. Sci.* **2020**, *21*, 4253; https://doi.org/10.3390/ijms21124253 (Ref. **54** in the References list to the given article)

137. Diana Anghel, Anca Lascu, Camelia Epuran, Ion Fratilescu, Catalin Ianasi, Mihaela Birdeanu and Eugenia Fagadar-Cosma. Hybrid Materials Based on Silica Matrices Impregnated with Pt-Porphyrin or PtNPs Destined for CO_2 Gas Detection or for Wastewaters Color Removal. *Int. J. Mol. Sci.* **2020**, *21*, 4262; https://doi.org/10.3390/ijms21124262 (Ref. **270** in the References list to the given article)

138. Zsolt Preisz, Zoltán Nagymihály, Beáta Lemli, László Kollár and Sándor Kunsági-Máté. Weak Interaction of the Antimetabolite Drug Methotrexate with a Cavitand Derivative. *Int. J. Mol. Sci.* **2020**, *21*, 4345; https://doi.org/10.3390/ijms21124345 (Ref. **7** in the References list to the given article)

139. Mario Gutiérrez, Lucie Duplouy-Armani, Lorenzo Angiolini, Mercedes Pintado-Sierra, Félix Sánchez and Abderrazzak Douhal. Femto- to Millisecond Time-Resolved Photodynamics of a Double-Functionalized Push–Pull Organic Linker: Potential Candidate for Optoelectronically Active MOFs. *Int. J. Mol. Sci.* **2020**, *21*, 4366; https://doi.org/10.3390/ijms21124366 (Ref. **146** in the References list to the given article)

140. Malcolm J. D'Souza, Jeremy Wirick, Osama Mahmoud, Dennis N. Kevill and Jin Burm Kyong. The Influence of a Terminal Chlorine Substituent on the Kinetics and the Mechanism of the Solvolyses of n-Alkyl Chloroformates in Hydroxylic Solvents. *Int. J. Mol. Sci.* **2020**, *21*, 4387; https://doi.org/10.3390/ijms21124387 (Ref. **42** in the References list to the given article)

141. Dylan Peukert, Ivan Kempson, Michael Douglass and Eva Bezak. Modelling Spatial Scales of Dose Deposition and Radiolysis Products from Gold Nanoparticle Sensitisation of Proton Therapy in A Cell: From Intracellular Structures to Adjacent Cells. *Int. J. Mol. Sci.* **2020**, *21*, 4431; https://doi.org/10.3390/ijms21124431 (Ref. **81** in the References list to the given article)

142. Magdalena Barwiolek, Anna Kaczmarek-Kędziera, Tadeusz M. Muziol, Dominika Jankowska, Julia Jezierska and Alina Bieńko. Dinuclear Copper(II) Complexes with Schiff Bases Derived from 2-Hydroxy-5-Methylisophthalaldehyde and Histamine or 2-(2-Aminoethyl)pyridine and Their Application as Magnetic and Fluorescent Materials in Thin Film Deposition. *Int. J. Mol. Sci.* **2020**, *21*, 4587; https://doi.org/10.3390/ijms21134587 (Ref. **147** in the References list to the given article)

143. Raj Kumar, Young Kyu Lee and Yong Seok Jho. Martini Coarse-Grained Model of Hyaluronic Acid for the Structural Change of Its Gel in the Presence of Monovalent and Divalent Salts. *Int. J. Mol. Sci.* **2020**, *21*, 4602; https://doi.org/10.3390/ijms21134602 (Ref. **148** in the References list to the given article)

144. Sarah A. Overall, Lauren E. Price, Brice J. Albert, Chukun Gao, Nicholas Alaniva, Patrick T. Judge, Erika L. Sesti, Paul A. Wender, George B. Kyei and Alexander B. Barnes. In Situ Detection of Endogenous HIV Activation by Dynamic Nuclear Polarization NMR and Flow Cytometry. *Int. J. Mol. Sci.* **2020**, *21*, 4649; https://doi.org/10.3390/ijms21134649 (Ref. **149** in the References list to the given article)

145. Patrice Porion and Alfred Delville. A Multi-Scale Study of Water Dynamics under Confinement, Exploiting Numerical Simulations in Relation to NMR Relaxometry, PGSE and NMR Micro-Imaging Experiments: An Application to the Clay/Water Interface. *Int. J. Mol. Sci.* **2020**, *21*, 4697; https://doi.org/10.3390/ijms21134697 (Ref. **242** in the References list to the given article)

146. Zhennan Zhao, Tingting Huang and Jiazhong Li. Molecular Dynamics Simulations to Investigate How PZM21 Affects the Conformational State of the μ-Opioid Receptor Upon Activation. *Int. J. Mol. Sci.* **2020**, *21*, 4699; https://doi.org/10.3390/ijms21134699 (Ref. **150** in the References list to the given article)

147. Tsuyoshi Murata, Taro Koide, Hirofumi Nobukuni, Ryotaro Tsuji and Yasushi Morita. 2D Coordination Network of Trioxotriangulene with Multiple Redox Abilities and Its Rechargeable Battery Performance. Int. *Int. J. Mol. Sci.* **2020**, *21*, 4723; https://doi.org/10.3390/ijms21134723 (Ref. **271** in the References list to the given article)

148. José Pedro Cerón-Carrasco. Theoretical Prediction of Dual-Potency Anti-Tumor Agents: Combination of Oxoplatin with Other FDA-Approved Oncology Drugs. *Int. J. Mol. Sci.* **2020**, *21*, 4741; https://doi.org/10.3390/ijms21134741 (Ref. **272** in the References list to the given article)

149. Lucas Sousa Martins, Jerônimo Lameira, Hendrik G. Kruger, Cláudio Nahum Alves and José Rogério A. Silva. Evaluating the Performance of a Non-Bonded Cu2+ Model Including Jahn−Teller Effect into the Binding of Tyrosinase Inhibitors. *Int. J. Mol. Sci.* **2020**, *21*, 4783; https://doi.org/10.3390/ijms21134783 (Ref. **202** in the References list to the given article)

150. Jiří Czernek and Jiří Brus. Polymorphic Forms of Valinomycin Investigated by NMR Crystallography. Int. *Int. J. Mol. Sci.* **2020**, *21*, 4907; https://doi.org/10.3390/ijms21144907 (Ref. **151** in the References list to the given article)

151. Samuel Zapién-Castillo, Nancy P. Díaz-Zavala, José A. Melo-Banda, Duncan Schwaller, Jean-Philippe Lamps, Marc Schmutz, Jérôme Combet and Philippe J. Mésini. Structure of Nanotubes Self-Assembled from a Monoamide Organogelator. *Int. J. Mol. Sci.* **2020**, *21*, 4960; https://doi.org/10.3390/ijms21144960 (Ref. **273** in the References list to the given article)

152. Debashis Majhi, Andrei V. Komolkin and Sergey V. Dvinskikh. NMR Spectroscopic Studies of Cation Dynamics in Symmetrically-Substituted Imidazolium-Based Ionic Liquid Crystals. *Int. J. Mol. Sci.* **2020**, *21*, 5024; https://doi.org/10.3390/ijms21145024 (Ref. **8** in the References list to the given article)

153. Daniel C. Elton, Peter D. Spencer, James D. Riches and Elizabeth D. Williams. Exclusion Zone Phenomena in Water—A Critical Review of Experimental Findings and Theories. *Int. J. Mol. Sci.* **2020**, *21*, 5041; https://doi.org/10.3390/ijms21145041 (Ref. **9** in the References list to the given article)

154. Marco Mendozza, Arianna Balestri, Costanza Montis and Debora Berti. Controlling the Kinetics of an Enzymatic Reaction through Enzyme or Substrate Confinement into Lipid Mesophases with Tunable Structural Parameters. *Int. J. Mol. Sci.* **2020**, *21*, 5116; https://doi.org/10.3390/ijms21145116 (Ref. **43** in the References list to the given article)

155. Navid Rabiee, Mojtaba Bagherzadeh, Amir Ghasemi, Hossein Zare, Sepideh Ahmadi, Yousef Fatahi, Rassoul Dinarvand, Mohammad Rabiee, Seeram Ramakrishna, Mohammadreza Shokouhimehr and Rajender S. Varma. Point-of-Use Rapid Detection of SARS-CoV-2: Nanotechnology-Enabled Solutions for the COVID-19 Pandemic. *Int. J. Mol. Sci.* **2020**, *21*, 5126; https://doi.org/10.3390/ijms21145126 (Ref. **274** in the References list to the given article)

156. Katarzyna Bucholc, Aleksandra Skrajna, Kinga Adamska, Xiao-Cui Yang, Krzysztof Krajewski, Jarosław Poznański, Michał Dadlez, Zbigniew Domiński and Igor Zhukov. Structural Analysis of the SANT/Myb Domain of FLASH and YARP Proteins and Their Complex with the C-Terminal Fragment of NPAT by NMR Spectroscopy and Computer Simulations. *Int. J. Mol. Sci.* **2020**, *21*, 5268; https://doi.org/10.3390/ijms21155268 (Ref. **152** in the References list to the given article)

157. Anna Nowik-Zajac, Iwona Zawierucha and Cezary Kozlowski. Selective Transport of Ag(I) through a Polymer Inclusion Membrane Containing a Calix[4]pyrrole Derivative from Nitrate Aqueous Solutions. Int. J. Mol. Sci. 2020, 21(15), 5348; https://doi.org/10.3390/ijms21155348 (Ref. **82** in the References list to the given article)

158. Aleksandra A. Ageeva, Simon V. Babenko, Ilya M. Magin, Victor F. Plyusnin, Polina S. Kuznetsova, Alexander A. Stepanov, Sergey F. Vasilevsky, Nikolay E. Polyakov, Alexander B. Doktorov and Tatyana V. Leshina. Stereoselectivity of Electron and Energy Transfer in the Quenching of (S/R)-Ketoprofen-(S)-Tryptophan Dyad Excited State. *Int. J. Mol. Sci.* **2020**, *21*, 5370; https://doi.org/10.3390/ijms21155370 (Ref. **243** in the References list to the given article)

159. Kazutami Sakamoto, Taku Morishita, Kenichi Aburai, Kenichi Sakai, Masahiko Abe, Ikuhiko Nakase, Shiroh Futaki and Hideki Sakai. Key Process and Factors Controlling the Direct Translocation of Cell-Penetrating Peptide through Bio-Membrane. *Int. J. Mol. Sci.* **2020**, *21*, 5466; https://doi.org/10.3390/ijms21155466 (Ref. **83** in the References list to the given article)

160. Agnieszka Chrzanowska, Anna Derylo-Marczewska and Malgorzata Wasilewska. Mesocellular Silica Foams (MCFs) with Tunable Pore Size as a Support for Lysozyme Immobilization: Adsorption Equilibrium and Kinetics, Biocomposite Properties. *Int. J. Mol. Sci.* **2020**, *21*, 5479; https://doi.org/10.3390/ijms21155479 (Ref. **275** in the References list to the given article)

161. Irina Stenina, Daniel Golubenko, Victor Nikonenko and Andrey Yaroslavtsev. Selectivity of Transport Processes in Ion-Exchange Membranes: Relationship with the Structure and Methods for Its Improvement. *Int. J. Mol. Sci.* **2020**, *21*, 5517; https://doi.org/10.3390/ijms21155517 (Ref. **84** in the References list to the given article)

162. Anita Adamoczky, Lajos Nagy, Miklós Nagy, Miklós Zsuga and Sándor Kéki. Conversion of Isocyanide to Amine in The Presence of Water and Hg(II) Ions: Kinetics and Mechanism as Detected by Fluorescence Spectroscopy and Mass Spectrometry. *Int. J. Mol. Sci.* **2020**, *21*, 5588; https://doi.org/10.3390/ijms21155588 (Ref. **44** in the References list to the given article)

163. Dariusz Karcz, Arkadiusz Matwijczuk, Daniel Kamiński, Bernadette Creaven,Ewa Ciszkowicz, Katarzyna Lecka-Szlachta and Karolina Starzak. Structural Features of 1,3,4-Thiadiazole-Derived Ligands and Their Zn(II) and Cu(II) Complexes Which Demonstrate Synergistic Antibacterial Effects with Kanamycin. *Int. J. Mol. Sci.* **2020**, *21*, 5735; https://doi.org/10.3390/ijms21165735 (Ref. **153** in the References list to the given article)

164. Hrvoje Rimac, Tana Tandarić, Robert Vianello and Mirza Bojić. Indomethacin Increases Quercetin Affinity for Human Serum Albumin: A Combined Experimental and Computational Study and Its Broader

Implications. *Int. J. Mol. Sci.* **2020**, *21*, 5740; https://doi.org/10.3390/ijms21165740 (Ref. **203** in the References list to the given article)

165. Verônica Muniz Couto, Laura de Oliveira-Nascimento, Luiz Fernando Cabeça, Danilo Costa Geraldes, Juliana Souza Ribeiro Costa, Karin A. Riske, Michelle Franz-Montan, Fabiano Yokaychiya, Margareth K.K. Dias Franco and Eneida de Paula. Capsaicin-Cyclodextrin Complex Enhances Mepivacaine Targeting and Improves Local Anesthesia in Inflamed Tissues. *Int. J. Mol. Sci.* **2020**, *21*, 5741; https://doi.org/10.3390/ijms21165741 (Ref. **154** in the References list to the given article)

166. Md Mofasserul Alam, Yaoming Wang, Chenxiao Jiang, Tingting Xu, Yahua Liu and Tongwen Xu. A Novel Anion Exchange Membrane for Bisulfite Anion Separation by Grafting a Quaternized Moiety through BPPO via Thermal-Induced Phase Separation. *Int. J. Mol. Sci.* **2020**, *21*, 5782; https://doi.org/10.3390/ijms21165782 (Ref. **85** in the References list to the given article)

167. Katarzyna Łudzik, Sebastian Woloszczuk, Wojciech Zając, Monika Jazdzewska, Andrey Rogachev, Alexander Ivanowicz Kuklin, Anna Zawisza and Małgorzata Jóźwiak. Can the Isothermal Calorimetric Curve Shapes Suggest the Structural Changes in Micellar Aggregates? *Int. J. Mol. Sci.* **2020**, *21*, 5828; https://doi.org/10.3390/ijms21165828 (Ref. **155** in the References list to the given article)

168. Zhen-Li Qi, Yun-Hui Cheng, Zhou Xu and Mao-Long Chen. Recent Advances in Porphyrin-Based Materials for Metal Ions Detection. *Int. J. Mol. Sci.* **2020**, *21*, 5839; https://doi.org/10.3390/ijms21165839 (Ref. **276** in the References list to the given article)

169. Agnieszka Tomczyk, Zofia Sokołowska, Patrycja Boguta and Katarzyna Szewczuk-Karpisz. Comparison of Monovalent and Divalent Ions Removal from Aqueous Solutions Using Agricultural Waste Biochars Prepared at Different Temperatures—Experimental and Model Study. *Int. J. Mol. Sci.* **2020**, *21*, 5851; https://doi.org/10.3390/ijms21165851 (Ref. **277** in the References list to the given article)

170. Su Young Jung, Dong Choon Park, Sung Su Kim and Seung Geun Yeo. Expression, Distribution and Role of Aquaporins in Various Rhinologic Conditions. *Int. J. Mol. Sci.* **2020**, *21*, 5853; https://doi.org/10.3390/ijms21165853 (Ref. **86** in the References list to the given article)

171. Martin Kos, Jaroslav Žádný, Jan Storch, Vladimír Církva, Petra Cuřínová, Jan Sýkora, Ivana Císařová, Febin Kuriakose and Igor. V. Alabugin. Oxidative Photocyclization of Aromatic Schiff Bases in Synthesis of Phenanthridines and Other Aza-PAHs. *Int. J. Mol. Sci.* **2020**, *21*, 5868; https://doi.org/10.3390/ijms21165868 (Ref. **156** in the References list to the given article)

172. M. A. Izquierdo-Gil, J. P. G. Villaluenga, S. Muñoz and V. M. Barragán. The Correlation between the Water Content and Electrolyte Permeability of Cation-Exchange Membranes. *Int. J. Mol. Sci.* **2020**, *21*, 5897; https://doi.org/10.3390/ijms21165897 (Ref. **87** in the References list to the given article)

173. Suhita Basumallick, Sourav Pal and Mihai V. Putz. Fock-Space Coupled Cluster Theory: Systematic Study of Partial Fourth Order Triples Schemes for Ionization Potential and Comparison with Bondonic Formalism. *Int. J. Mol. Sci.* **2020**, *21*, 6199; https://doi.org/10.3390/ijms21176199 (Ref. **204** in the References list to the given article)

174. Veronika Zeindlhofer, Phillip Hudson, Ádám Márk Pálvölgyi, Matthias Welsch, Mazin Almarashi, H. Lee Woodcock, Bernard Brooks, Katharina Bica-Schröder and Christian Schröder. Enantiomerization of Axially Chiral Biphenyls: Polarizable MD Simulations in Water and Butylmethylether. *Int. J. Mol. Sci.* **2020**, *21*, 6222; https://doi.org/10.3390/ijms21176222 (Ref. **205** in the References list to the given article)

175. Jiaoxue Yang, Guochun Lv, Chenxi Zhang, Zehua Wang and Xiaomin Sun. Indirect Photodegradation of Sulfamethoxazole and Trimethoprim by Hydroxyl Radicals in Aquatic Environment: Mechanisms, Transformation Products and Eco-Toxicity Evaluation. *Int. J. Mol. Sci.* **2020**, *21*, 6276; https://doi.org/10.3390/ijms21176276 (Ref. **206** in the References list to the given article)

176. Wojciech Kujawski, Andriy Yaroshchuk, Emiliy Zholkovskiy, Izabela Koter and Stanislaw Koter. Analysis of Membrane Transport Equations for Reverse Electrodialysis (RED) Using Irreversible Thermodynamics. *Int. J. Mol. Sci.* **2020**, *21*, 6325; https://doi.org/10.3390/ijms21176325 (Ref. **88** in the References list to the given article)

177. Paulina Spisz, Witold Kozak, Lidia Chomicz-Mańka, Samanta Makurat, Karina Falkiewicz, Artur Sikorski, Anna Czaja, Janusz Rak and Magdalena Zdrowowicz. 5-(N-Trifluoromethylcarboxy)aminouracil as a Potential DNA Radiosensitizer and Its Radiochemical Conversion into N-Uracil-5-yloxamic Acid. *Int. J. Mol. Sci.* **2020**, *21*, 6352; https://doi.org/10.3390/ijms21176352 (Ref. **207** in the References list to the given article)

178. Jarrod Schiffbauer, Evgeny Demekhin and Georgy Ganchenko. Transitions and Instabilities in Imperfect Ion-Selective Membranes. *Int. J. Mol. Sci.* **2020**, *21*, 6526; https://doi.org/10.3390/ijms21186526 (Ref. **90** in the References list to the given article)

179. Kelvin Suggs and Alfred Z. Msezane. Doubly-Charged Negative Ions as Novel Tunable Catalysts: Graphene and Fullerene Molecules Versus Atomic Metals. *Int. J. Mol. Sci.* **2020**, *21*, 6714; https://doi.org/10.3390/ijms21186714181 (Ref. **208** in the References list to the given article)

180. Lai Yee Lee, Norhashimah Morad, Norli Ismail, Amir Talebi and Mohd Rafatullah. Optimization for Liquid-Liquid Extraction of Cd(II) over Cu(II) Ions from Aqueous Solutions Using Ionic Liquid Aliquat 336 with Tributyl Phosphate. *Int. J. Mol. Sci.* **2020**, *21*, 6860; https://doi.org/10.3390/ijms21186860 (Ref. **10** in the References list to the given article)

181. Anton Muravev, Tatiana Gerasimova, Robert Fayzullin, Olga Babaeva, Ildar Rizvanov, Ayrat Khamatgalimov, Marsil Kadirov, Sergey Katsyuba, Igor Litvinov, Shamil Latypov, Svetlana Solovieva and Igor Antipin. Thermally Stable Nitrothiacalixarene Chromophores: Conformational Study and Aggregation Behavior. *Int. J. Mol. Sci.* **2020**, *21*, 6916; https://doi.org/10.3390/ijms21186916 (Ref. **157** in the References list to the given article)

182. Kamila Butowska, Krzysztof Żamojć, Mateusz Kogut, Witold Kozak, Dariusz Wyrzykowski, Wiesław Wiczk, Jacek Czub, Jacek Piosik and Janusz Rak. The Product of Matrix Metalloproteinase Cleavage of Doxorubicin Conjugate for Anticancer Drug Delivery: Calorimetric, Spectroscopic, and Molecular Dynamics Studies on Peptide–Doxorubicin Binding to DNA. *Int. J. Mol. Sci.* **2020**, *21*, 6923; https://doi.org/10.3390/ijms21186923 (Ref. **278** in the References list to the given article)

183. Filipe Costa, Ali Traoré-Dubuis, Lidia Álvarez, Ana I. Lozano, Xueguang Ren, Alexander Dorn, Paulo Limão-Vieira, Francisco Blanco, Juan C. Oller, Antonio Muñoz, Adrián García-Abenza, Jimena D. Gorfinkiel, Alessandra S. Barbosa, Marcio H.F. Bettega, Peter Stokes, Ronald D. White, Darryl B. Jones, Michael J. Brunger and Gustavo García. A Complete Cross Section Data Set for Electron Scattering by Pyridine: Modelling Electron Transport in the Energy Range 0–100 eV. *Int. J. Mol. Sci.* **2020**, *21*, 6947; https://doi.org/10.3390/ijms21186947 (Ref. **244** in the References list to the given article)

184. Sirinuch Nanthapong, Soorathep Kheawhom and Chalida Klaysom. MCM-41/PVA Composite as a Separator for Zinc–Air Batteries. *Int. J. Mol. Sci.* **2020**, *21*, 7052; https://doi.org/10.3390/ijms21197052 (Ref. **90** in the References list to the given article)

185. Taku Shoji, Tetsuo Okujima and Shunji Ito. Development of Heterocycle-Substituted and Fused Azulenes in the Last Decade (2010–2020). *Int. J. Mol. Sci.* **2020**, *21*, 7087; https://doi.org/10.3390/ijms21197087 (Ref. **158** in the References list to the given article)

186. Mainak Karmakar, Antonio Frontera, Shouvik Chattopadhyay, Tiddo J. Mooibroek and Antonio Bauzá. Intramolecular Spodium Bonds in Zn(II) Complexes: Insights from Theory and Experiment. *Int. J. Mol. Sci.* **2020**, *21*, 7091; https://doi.org/10.3390/ijms21197091 (Ref. **209** in the References list to the given article)

187. Hosung Ki, Sungjun Park, Seunghwan Eom, Jain Gu, Siin Kim, Changwon Kim, Chi Woo Ahn, Minseo Choi, Sena Ahn, Doo-Sik Ahn, Jungkweon Choi, Mu-Hyun Baik and Hyotcherl Ihee. Gold Nanoparticle Formation via X-ray Radiolysis Investigated with Time-Resolved X-ray Liquidography. *Int. J. Mol. Sci.* **2020**, *21*, 7125; https://doi.org/10.3390/ijms21197125 (Ref. **159** in the References list to the given article)

188. Francesco Mallamace, Giuseppe Mensitieri, Domenico Mallamace, Martina Salzano de Luna and Sow-Hsin Chen. Some Aspects of the Liquid Water Thermodynamic Behavior: From The Stable to the Deep Supercooled Regime. *Int. J. Mol. Sci.* **2020**, *21*, 7269; https://doi.org/10.3390/ijms21197269 (Ref. **11** in the References list to the given article)

189. Alexander S. Paramonov, Milita V. Kocharovskaya, Andrey V. Tsarev, Dmitrii S. Kulbatskii, Eugene V. Loktyushov, Mikhail A. Shulepko, Mikhail P. Kirpichnikov, Ekaterina N. Lyukmanova and Zakhar O. Shenkarev. Structural Diversity and Dynamics of Human Three-Finger Proteins Acting on Nicotinic Acetylcholine Receptors. *Int. J. Mol. Sci.* **2020**, *21*, 7280; https://doi.org/10.3390/ijms21197280 (Ref. **160** in the References list to the given article)

190. Ramin Khezri, Soraya Hosseini, Abhishek Lahiri, Shiva Rezaei Motlagh, Mai Thanh Nguyen, Tetsu Yonezawa and Soorathep Kheawhom. Enhanced Cycling Performance of Rechargeable Zinc–Air Flow Batteries Using Potassium Persulfate as Electrolyte Additive. *Int. J. Mol. Sci.* **2020**, *21*, 7303; https://doi.org/10.3390/ijms21197303 (Ref. **245** in the References list to the given article)

191. Kazutami Sakamoto, Takeshi Kitano, Haruka Kuwahara, Megumi Tedani, Kenichi Aburai, Shiroh Futaki, Masahiko Abe, Hideki Sakai, Hiroyasu Ohtaka and Yuji Yamashita. Effect of Vesicle Size on the Cytolysis of Cell-Penetrating Peptides (CPPs). *Int. J. Mol. Sci.* **2020**, *21*, 7405; https://doi.org/10.3390/ijms21197405 (Ref. **91** in the References list to the given article)

192. Grigorii S. Bocharov and Alexander V. Eletskii. Percolation Conduction of Carbon Nanocomposites. *Int. J. Mol. Sci.* **2020**, *21*, 7634; https://doi.org/10.3390/ijms21207634 (Ref. **279** in the References list to the given article)

193. Mona Koder Hamid, Axel Rüter, Stefan Kuczera and Ulf Olsson. Slow Dissolution Kinetics of Model Peptide Fibrils. *Int. J. Mol. Sci.* **2020**, *21*, 7671; https://doi.org/10.3390/ijms21207671 (Ref. **45** in the References list to the given article)

194. Wagner Silva, Marcileia Zanatta, Ana Sofia Ferreira, Marta C. Corvo and Eurico J. Cabrita. Revisiting Ionic Liquid Structure-Property Relationship: A Critical Analysis. *Int. J. Mol. Sci.* **2020**, *21*, 7745; https://doi.org/10.3390/ijms21207745 (Ref. **55** in the References list to the given article)

195. Mohamed Alae Ait Kerroum, Cristian Iacovita, Walid Baaziz, Dris Ihiawakrim, Guillaume Rogez, Mohammed Benaissa, Constantin Mihai Lucaciu and Ovidiu Ersen. Quantitative Analysis of the Specific Absorption Rate Dependence on the Magnetic Field Strength in $Zn_xFe_{3-x}O_4$ Nanoparticles. *Int. J. Mol. Sci.* **2020**, *21*, 7775; https://doi.org/10.3390/ijms21207775 (Ref. **280** in the References list to the given article)

196. Tomislav Jednačak, Ivana Mikulandra and Predrag Novak. Advanced Methods for Studying Structure and Interactions of Macrolide Antibiotics. *Int. J. Mol. Sci.* **2020**, *21*, 7799; https://doi.org/10.3390/ijms21207799 (Ref. **161** in the References list to the given article)

197. Sabina Brazevic, Stanisław Nizinski, Michel Sliwa, Jiro Abe, Michał F. Rode and Gotard Burdzinski. Control of the Photo-Isomerization Mechanism in 3H-Naphthopyrans to Prevent Formation of Unwanted Long-Lived Photoproducts. *Int. J. Mol. Sci.* **2020**, *21*, 7825; https://doi.org/10.3390/ijms21217825 (Ref. **162** in the References list to the given article)

198. Natalia Pismenskaya, Veronika Sarapulova, Anastasia Klevtsova, Sergey Mikhaylin and Laurent Bazinet. Adsorption of Anthocyanins by Cation and Anion Exchange Resins with Aromatic and Aliphatic Polymer Matrices. *Int. J. Mol. Sci.* **2020**, *21*, 7874; https://doi.org/10.3390/ijms21217874 (Ref. **92** in the References list to the given article)

199. Luca Rigamonti, Paolo Zardi, Stefano Carlino, Francesco Demartin, Carlo Castellano, Laura Pigani, Alessandro Ponti, Anna Maria Ferretti and Alessandro Pasini. Selective Formation, Reactivity, Redox and Magnetic Properties of MnIII and FeIII Dinuclear Complexes with Shortened Salen-Type Schiff Base Ligands. *Int. J. Mol. Sci.* **2020**, *21*, 7882; https://doi.org/10.3390/ijms21217882 (Ref. **163** in the References list to the given article)

200. Rafael R. Castillo, Lorena de la Torre, Félix García-Ochoa, Miguel Ladero and María Vallet-Regí. Production of MCM-41 Nanoparticles with Control of Particle Size and Structural Properties: Optimizing Operational Conditions during Scale-Up. *Int. J. Mol. Sci.* **2020**, *21*, 7899; https://doi.org/10.3390/ijms21217899 (Ref. **12** in the References list to the given article)

201. Jiří Czernek and Jiří Brus. Parametrizing the Spatial Dependence of 1H NMR Chemical Shifts in π-Stacked Molecular Fragments. *Int. J. Mol. Sci.* **2020**, *21*, 7908; https://doi.org/10.3390/ijms21217908 (Ref. **210** in the References list to the given article)

202. Sebok Lee, Myungsam Jen and Yoonsoo Pang. Twisted Intramolecular Charge Transfer State of a "Push-Pull" Emitter *Int. J. Mol. Sci.* **2020**, *21*, 7999; https://doi.org/10.3390/ijms21217999 (Ref. **211** in the References list to the given article)

203. Changsun Eun. Osmosis-Driven Water Transport through a Nanochannel: A Molecular Dynamics Simulation Study. *Int. J. Mol. Sci.* **2020**, *21*, 8030; https://doi.org/10.3390/ijms21218030 (Ref. **13** in the References list to the given article)

204. Sergey V. Gudkov, Nikita V. Penkov, Ilya V. Baimler, Gennady A. Lyakhov, Vladimir I. Pustovoy, Alexander V. Simakin, Ruslan M. Sarimov and Ivan A. Scherbakov. Effect of Mechanical Shaking on the Physicochemical Properties of Aqueous Solutions. *Int. J. Mol. Sci.* **2020**, *21*, 8033; https://doi.org/10.3390/ijms21218033 (Ref. **14** in the References list to the given article)

205. Ibon Alkorta, José Elguero, Cristina Trujillo and Goar Sánchez-Sanz. Interaction between Trinuclear Regium Complexes of Pyrazolate and Anions, a Computational Study. *Int. J. Mol. Sci.* **2020**, *21*, 8036; https://doi.org/10.3390/ijms21218036 (Ref. **212** in the References list to the given article)

206. Songlin Xue, Daiki Kuzuhara, Naoki Aratani and Hiroko Yamada. Vinylene-Bridged Cyclic Dipyrrin and BODIPY Trimers. *Int. J. Mol. Sci.* **2020**, *21*, 8041; https://doi.org/10.3390/ijms21218041 (Ref. **164** in the References list to the given article)

207. Kenji Aramaki, Eriko Takimoto and Takumi Yamaguchi. Effect of the Cationic Head Group on Cationic Surfactant-Based Surfactant Mediated Gelation (SMG). *Int. J. Mol. Sci.* **2020**, *21*, 8046; https://doi.org/10.3390/ijms21218046 (Ref. **165** in the References list to the given article)

208. Fioretta Asaro, Carla Boga, Rita De Zorzi, Silvano Geremia, Lara Gigli, Patrizia Nitti and Sabrina Semeraro. (R)-10-Hydroxystearic Acid: Crystals vs. Organogel. *Int. J. Mol. Sci.* **2020**, *21*, 8124; https://doi.org/10.3390/ijms21218124 (Ref. **166** in the References list to the given article)

209. Magdalena Laskowska, Oleksandr Pastukh, Andrii Fedorchuk, Mateusz Schabikowski, Paweł Kowalczyk, Marcin Zalasiński and Łukasz Laskowski. Nanostructured Silica with Anchoring Units: The 2D Solid Solvent for Molecules and Metal Ions. *Int. J. Mol. Sci.* **2020**, *21*, 8137; https://doi.org/10.3390/ijms21218137 (Ref. **281** in the References list to the given article)

210. Thomas F. M. Luxford, Stanislav A. Pshenichnyuk, Nail L. Asfandiarov, Tomáš Perečko, Martin Falk and Jaroslav Kočišek. 5-Nitro-2,4-Dichloropyrimidine as an Universal Model for Low-Energy Electron Processes Relevant for Radiosensitization. *Int. J. Mol. Sci.* **2020**, *21*, 8173; https://doi.org/10.3390/ijms21218173 (Ref. **229** in the References list to the given article)

211. Rinaldo Grazioso, Sara García-Viñuales, Luigi Russo, Gianluca D'Abrosca, Sabrina Esposito, Laura Zaccaro, Rosa Iacovino, Danilo Milardi, Roberto Fattorusso, Gaetano Malgieri and Carla Isernia. Substitution of the Native Zn(II) with Cd(II), Co(II) and Ni(II) Changes the Downhill Unfolding Mechanism of Ros87 to a Completely Different Scenario. *Int. J. Mol. Sci.* **2020**, *21*, 8285; https://doi.org/10.3390/ijms21218285 (Ref. **167** in the References list to the given article)

212. María Montes-Casado, Adrian Sanvicente, Laura Casarrubios, María José Feito, José M. Rojo, María Vallet-Regí, Daniel Arcos, Pilar Portolés and María Teresa Portolés. An Immunological Approach to the Biocompatibility of Mesoporous SiO_2-CaO Nanospheres. *Int. J. Mol. Sci.* **2020**, *21*, 8291; https://doi.org/10.3390/ijms21218291 (Ref. **15** in the References list to the given article)

213. Shao-Lun Lu, Wei-Wen Liu, Jason Chia-Hsien Cheng, Lien-Chieh Lin, Churng-Ren Chris Wang and Pai-Chi Li. Enhanced Radiosensitization for Cancer Treatment with Gold Nanoparticles through Sonoporation. *Int. J. Mol. Sci.* **2020**, *21*, 8370; https://doi.org/10.3390/ijms21218370 (Ref. **246** in the References list to the given article)

214. Soyoung Hwang, Dong Min Shin and Jeong Hee Hong. Intracellular Ca2+-Mediated AE2 Is Involved in the Vectorial Movement of HaCaT Keratinocyte. *Int. J. Mol. Sci.* **2020**, *21*, 8429; https://doi.org/10.3390/ijms21228429 (Ref. **93** in the References list to the given article)

215. Vanessa Poscher, George S. Pappas, Oliver Brüggemann, Ian Teasdale and Yolanda Salinas. Hybrid Porous Microparticles Based on a Single Organosilica Cyclophosphazene Precursor. *Int. J. Mol. Sci.* **2020**, *21*, 8552; https://doi.org/10.3390/ijms21228552 (Ref. **16** in the References list to the given article)

216. Marco Savioli,Manuela Stefanelli, Gabriele Magna,Francesca Zurlo, Maria Federica Caso, Rita Cimino, Claudio Goletti, Mariano Venanzi, Corrado Di Natale, Roberto Paolesse and Donato Monti. Tunable Supramolecular Chirogenesis in the Self-Assembling of Amphiphilic Porphyrin Triggered by Chiral Amines. *Int. J. Mol. Sci.* **2020**, *21*, 8557; https://doi.org/10.3390/ijms21228557 (Ref. **168** in the References list to the given article)

217. Carina Karner, Felix Müller and Emanuela Bianchi. A Matter of Size and Placement: Varying the Patch Size of Anisotropic Patchy Colloids. *Int. J. Mol. Sci.* **2020**, *21*, 8621; https://doi.org/10.3390/ijms21228621 (Ref. **17** in the References list to the given article)

218. Adam Sułek, Barbara Pucelik, Marcin Kobielusz, Agata Barzowska and Janusz M. Dąbrowski. Photodynamic Inactivation of Bacteria with Porphyrin Derivatives: Effect of Charge, Lipophilicity, ROS Generation, and Cellular Uptake on Their Biological Activity In Vitro. *Int. J. Mol. Sci.* **2020**, *21*, 8716; https://doi.org/10.3390/ijms21228716 (Ref. **169** in the References list to the given article)

219. Abderrahim Titi, Mouslim Messali, Rachid Touzani, Mohammed Fettouhi, Abdelkader Zarrouk, Nabil Al-Zaqri, Ali Alsalme, Fahad A. Alharthi, Amjad Alsyahi and Ismail Warad. Synthesis of Novel Tetra(μ3-Methoxo) Bridged with [Cu(II)-O-Cd(II)] Double-Open-Cubane Cluster: XRD/HSA-Interactions, Spectral and Oxidizing Properties. *Int. J. Mol. Sci.* **2020**, *21*, 8787; https://doi.org/10.3390/ijms21228787 (Ref. **170** in the References list to the given article)

220. Muhammad Saqib, Eugene Arthur-Baidoo, Milan Ončák and Stephan Denifl. Electron Attachment Studies with the Potential Radiosensitizer 2-Nitrofuran. *Int. J. Mol. Sci.* **2020**, *21*, 8906; https://doi.org/10.3390/ijms21238906 (Ref. **229** in the References list to the given article)

221. Lourdes Pérez, Aurora Pinazo, M.C. Morán and Ramon Pons. Aggregation Behavior, Antibacterial Activity and Biocompatibility of Catanionic Assemblies Based on Amino Acid-Derived Surfactants. *Int. J. Mol. Sci.* **2020**, *21*, 8912; https://doi.org/10.3390/ijms21238912 (Ref. **18** in the References list to the given article)

222. Anna V. Vologzhanina, Ivan E. Ushakov and Alexander A. Korlyukov. Intermolecular Interactions in Crystal Structures of Imatinib-Containing Compounds. *Int. J. Mol. Sci.* **2020**, *21*, 8970; https://doi.org/10.3390/ijms21238970 (Ref. **171** in the References list to the given article)

223. Oleg V. Mikhailov and Denis V. Chachkov. DFT Quantum-Chemical Modeling Molecular Structures of Cobalt Macrocyclic Complexes with Porphyrazine or Its Benzo-Derivatives and Two Oxygen Acido Ligands. *Int. J. Mol. Sci.* **2020**, *21*, 9085; https://doi.org/10.3390/ijms21239085 (Ref. **213** in the References list to the given article)

224. Irina Terekhova, Iliya Kritskiy, Mikhail Agafonov, Roman Kumeev, Carlos Martínez-Cortés and Horacio Pérez-Sánchez. Selective Binding of Cyclodextrins with Leflunomide and Its Pharmacologically Active Metabolite Teriflunomide. *Int. J. Mol. Sci.* **2020**, *21*, 9102; https://doi.org/10.3390/ijms21239102 (Ref. **19** in the References list to the given article)

225. Ilaria Passarini, Pedro Ernesto de Resende, Sarah Soares, Tadeh Tahmasi, Paul Stapleton, John Malkinson, Mire Zloh and Sharon Rossiter. Synthesis and in Silico Modelling of the Potential Dual Mechanistic Activity of Small Cationic Peptides Potentiating the Antibiotic Novobiocin against Susceptible and Multi-Drug Resistant Escherichia coli. *Int. J. Mol. Sci.* **2020**, *21*, 9134; https://doi.org/10.3390/ijms21239134 (Ref. **172** in the References list to the given article)

226. Ion Călina, Maria Demeter, Anca Scărișoreanu, Veronica Sătulu and Bogdana Mitu. One Step e-Beam Radiation Cross-Linking of Quaternary Hydrogels Dressings Based on Chitosan-Poly(Vinyl-Pyrrolidone)-Poly(Ethylene Glycol)-Poly(Acrylic Acid). *Int. J. Mol. Sci.* **2020**, *21*, 9236; https://doi.org/10.3390/ijms21239236 (Ref. **282** in the References list to the given article)

227. Abigail L. Barker, Hamlin Barnes and Franck E. Dayan. Conformation of the Intermediates in the Reaction Catalyzed by Protoporphyrinogen Oxidase: An In Silico Analysis. *Int. J. Mol. Sci.* **2020**, *21*, 9495; https://doi.org/10.3390/ijms21249495 (Ref. **173** in the References list to the given article)

228. Alfredo Fuentes-Gutiérrez, Everardo Curiel-Quesada, José Correa-Basurto, Alberto Martínez-Muñoz and Alicia Reyes-Arellano. N-Heterocycles Scaffolds as Quorum Sensing Inhibitors. Design, Synthesis, Biological and Docking Studies. *Int. J. Mol. Sci.* **2020**, *21*, 9512; https://doi.org/10.3390/ijms21249512 (Ref. **174** in the References list to the given article)

229. Ana Rodríguez-Ramos, Laura Marín-Caba, Nerea Iturrioz-Rodríguez, Esperanza Padín-González, Lorena García-Hevia, Teresa Mêna Oliveira, Miguel A. Corea-Duarte and Mónica L. Fanarraga. Design of Polymeric and Biocompatible Delivery Systems by Dissolving Mesoporous Silica Templates *Int. J. Mol. Sci.* **2020**, *21*, 9573; https://doi.org/10.3390/ijms21249573 (Ref. **20** in the References list to the given article)

230. Miguel Gisbert-Garzarán, Daniel Lozano and María Vallet-Regí. Mesoporous Silica Nanoparticles for Targeting Subcellular Organelles. *Int. J. Mol. Sci.* **2020**, *21*, 9696; https://doi.org/10.3390/ijms21249696 (Ref. **21** in the References list to the given article)

231. Franco Cataldo. On the Optical Activity of Poly(l-lactic acid) (PLLA) Oligomers and Polymer: Detection of Multiple Cotton Effect on Thin PLLA Solid Film Loaded with Two Dyes. *Int. J. Mol. Sci.* **2021**, *22*, 8; https://doi.org/10.3390/ijms22010008 (Ref. **283** in the References list to the given article)

232. Nadia Manganaro, Roberto Zagami, Mariachiara Trapani, Maria Angela Castriciano, Andrea Romeo and Luigi Monsù Scolaro. Role of Cobalt(III) Cationic Complexes in the Self-Assembling Process of a Water Soluble Porphyrin. *Int. J. Mol. Sci.* **2021**, *22*, 39; https://doi.org/10.3390/ijms22010039 (Ref. **175** in the References list to the given article)

233. Domenico Mallamace, Georgia Papanikolaou, Siglinda Perathoner, Gabriele Centi and Paola Lanzafame. Comparing Molecular Mechanisms in Solar NH_3 Production and Relations with CO_2 Reduction. *Int. J. Mol. Sci.* **2021**, *22*, 139; https://doi.org/10.3390/ijms22010139 (Ref. **247** in the References list to the given article)

234. Mihai V. Putz. Chemical Bonding by the Chemical Orthogonal Space of Reactivity. *Int. J. Mol. Sci.* **2021**, *22*, 223; https://doi.org/10.3390/ijms22010223 (Ref. **214** in the References list to the given article)

235. Luigi Santamaria, Valentina Di Sarno, Roberto Aiello, Maurizio De Rosa, Iolanda Ricciardi, Paolo De Natale and Pasquale Maddaloni. Infrared Comb Spectroscopy of Buffer-Gas-Cooled Molecules: Toward Absolute Frequency Metrology of Cold Acetylene. *Int. J. Mol. Sci.* **2021**, *22*, 250; https://doi.org/10.3390/ijms22010250 (Ref. **176** in the References list to the given article)

236. Elizabeth M. Timmers, Jose Rodrigo Magana, Sandra M.C. Schoenmakers, P. Michel Fransen, Henk M. Janssen and Ilja K. Voets. Sequence of Polyurethane Ionomers Determinative for Core Structure of Surfactant–Copolymer Complexes. *Int. J. Mol. Sci.* **2021**, *22*, 337; https://doi.org/10.3390/ijms22010337 (Ref. **177** in the References list to the given article)

237. Amani Zoabi and Katherine Margulis. Differential Interactions of Chiral Nanocapsules with DNA. *Int. J. Mol. Sci.* **2021**, *22*, 584; https://doi.org/10.3390/ijms22020584 (Ref. **284** in the References list to the given article)

238. Konrad Skotnicki, Slawomir Ostrowski, Jan Cz. Dobrowolski, Julio R. De la Fuente, Alvaro Cañete and Krzysztof Bobrowski. Spectral Probe for Electron Transfer and Addition Reactions of Azide Radicals with Substituted Quinoxalin-2-Ones in Aqueous Solutions. *Int. J. Mol. Sci.* **2021**, *22*, 633; https://doi.org/10.3390/ijms22020633 (Ref. **230** in the References list to the given article)

239. Jelena Vukalović, Jelena B. Maljković, Karoly Tökési, Branko Predojević and Bratislav P. Marinković. Elastic Electron Scattering from Methane Molecule in the Energy Range from 50–300 eV. *Int. J. Mol. Sci.* **2021**, *22*, 647; https://doi.org/10.3390/ijms22020647 (Ref. **248** in the References list to the given article)

240. Rui Cordeiro, Maria J. Beira, Carlos Cruz, João L. Figueirinhas, Marta C. Corvo, Pedro L. Almeida, Andreia A. Rosatella, Carlos A.M. Afonso, Carla I. Daniel and Pedro J. Sebastião. Tuning the 1H NMR Paramagnetic Relaxation Enhancement and Local Order of [Aliquat]+-Based Systems Mixed with DMSO. *Int. J. Mol. Sci.* **2021**, *22*, 706; https://doi.org/10.3390/ijms22020706 (Ref. **56** in the References list to the given article)

241. Giulia Rossella Delpiano, Davide Tocco, Luca Medda, Edmond Magner and Andrea Salis. Adsorption of Malachite Green and Alizarin Red S Dyes Using Fe-BTC Metal Organic Framework as Adsorbent. *Int. J. Mol. Sci.* **2021**, *22*, 788; https://doi.org/10.3390/ijms22020788 (Ref. **285** in the References list to the given article)

242. Maria Angela Castriciano, Sergio Cardillo, Roberto Zagami, Mariachiara Trapani, Andrea Romeo and Luigi Monsù Scolaro. Effects of the Mixing Protocol on the Self-Assembling Process of Water Soluble Porphyrins. *Int. J. Mol. Sci.* **2021**, *22*, 797; https://doi.org/10.3390/ijms22020797 (Ref. **178** in the References list to the given article)

243. Oleg V. Mikhailov. Preface from the Guest Editor of Special Issue "Simple Substances of Non-Metals: Molecular Structures Modeling with Using DFT and More Advanced Methods of Quantum Chemistry". *Int. J. Mol. Sci.* **2021**, *22*, 815; https://doi.org/10.3390/ijms22020815 (Ref. **215** in the References list to the given article)

244. Ionut Avramia and Sonia Amariei. Spent Brewer's Yeast as a Source of Insoluble β-Glucans. *Int. J. Mol. Sci.* **2021**, *22*, 825; https://doi.org/10.3390/ijms22020825 (Ref. **286** in the References list to the given article)

245. Francisco José Ostos, José Antonio Lebrón, María Luisa Moyá, Eva Bernal, Ana Flores, Cristian Lépori, Ángeles Maestre, Francisco Sánchez, Pilar López-Cornejo and Manuel López-López. Potentiometric Study of Carbon Nanotube/Surfactant Interactions by Ion-Selective Electrodes. Driving Forces in the Adsorption and Dispersion Processes. *Int. J. Mol. Sci.* **2021**, *22*, 826; https://doi.org/10.3390/ijms22020826 (Ref. **22** in the References list to the given article)

246. Krzysztof Żamojć, Irena Bylińska, Wiesław Wiczk and Lech Chmurzyński. Fluorescence Quenching Studies on the Interactions between Chosen Fluoroquinolones and Selected Stable TEMPO and PROXYL Nitroxides. *Int. J. Mol. Sci.* **2021**, *22*, 885; https://doi.org/10.3390/ijms22020885 (Ref. **249** in the References list to the given article)

247. Kenneth R. Olson, Yan Gao and Karl D. Straub. Oxidation of Hydrogen Sulfide by Quinones: How Polyphenols Initiate Their Cytoprotective Effects. *Int. J. Mol. Sci.* **2021**, *22*, 961; https://doi.org/10.3390/ijms22020961 (Ref. **46** in the References list to the given article)

248. Skadi Lau, Manfred Gossen, Andreas Lendlein and Friedrich Jung. Venous and Arterial Endothelial Cells from Human Umbilical Cords: Potential Cell Sources for Cardiovascular Research. *Int. J. Mol. Sci.* **2021**, *22*, 978; https://doi.org/10.3390/ijms22020978 (Ref. **94** in the References list to the given article)

249. Teng-Hui Wang, Li-Wen Hsu and Hai-Chou Chang. Structural Reorganization of Imidazolium Ionic Liquids Induced by Pressure-Enhanced Ionic Liquid—Polyethylene Oxide Interactions. *Int. J. Mol. Sci.* **2021**, *22*, 981; https://doi.org/10.3390/ijms22020981 (Ref. **57** in the References list to the given article)

250. Natalia Pawlik, Barbara Szpikowska-Sroka, Tomasz Goryczka and Wojciech A. Pisarski. Studies of Sol-Gel Evolution and Distribution of Eu3+ Ions in Glass–Ceramics Containing LaF3 Nanocrystals Depending on Initial Sols Composition. *Int. J. Mol. Sci.* **2021**, *22*, 996; https://doi.org/10.3390/ijms22030996 (Ref. **287** in the References list to the given article)

251. Shunji Yamada, Eisuke Chikayama and Jun Kikuchi. Signal Deconvolution and Generative Topographic Mapping Regression for Solid-State NMR of Multi-Component Materials. *Int. J. Mol. Sci.* **2021**, 22(3), 1086; https://doi.org/10.3390/ijms22031086 (Ref. **288** in the References list to the given article)

252. Antía Gonzalez Pereira, Maria Carpena, Paula García Oliveira, Juan Carlos Mejuto, Miguel Angel Prieto and Jesus Simal Gandara. Main Applications of Cyclodextrins in the Food Industry as the Compounds of Choice to Form Host–Guest Complexes. *Int. J. Mol. Sci.* **2021**, *22*, 1339; https://doi.org/10.3390/ijms22031339 (Ref. **289** in the References list to the given article)

253. V. V. Krishnan, Timothy Bentley, Alina Xiong and Kalyani Maitra. Conformational Ensembles by NMR and MD Simulations in Model Heptapeptides with Select Tri-Peptide Motifs. *Int. J. Mol. Sci.* **2021**, *22*, 1364; https://doi.org/10.3390/ijms22031364 (Ref. **179** in the References list to the given article)

254. Veronika Sarapulova, Natalia Pismenskaya, Valentina Titorova, Mikhail Sharafan, Yaoming Wang, Tongwen Xu, Yang Zhang and Victor Nikonenko. Transport Characteristics of CJMAED™ Homogeneous Anion Exchange Membranes in Sodium Chloride and Sodium Sulfate Solutions. *Int. J. Mol. Sci.* **2021**, *22*, 1415; https://doi.org/10.3390/ijms22031415 (Ref. **95** in the References list to the given article)

255. Petre Ionita. The Chemistry of DPPH· Free Radical and Congeners. *Int. J. Mol. Sci.* **2021**, *22*, 1545; https://doi.org/10.3390/ijms22041545 (Ref. **231** in the References list to the given article)

256. Malgorzata Latos-Brozio and Anna Masek. Environmentally Friendly Polymer Compositions with Natural Amber Acid. *Int. J. Mol. Sci.* **2021**, *22*, 1556; https://doi.org/10.3390/ijms22041556 (Ref. **290** in the References list to the given article)

257. Federica Banche-Niclot, Giorgia Montalbano, Sonia Fiorilli and Chiara Vitale-Brovarone. PEG-Coated Large Mesoporous Silicas as Smart Platform for Protein Delivery and Their Use in a Collagen-Based Formulation for 3D Printing. *Int. J. Mol. Sci.* **2021**, *22*, 1718; https://doi.org/10.3390/ijms22041718 (Ref. **96** in the References list to the given article)

258. Michael Bell, Anil Kumar and Michael D. Sevilla. Electron-Induced Repair of 2′-Deoxyribose Sugar Radicals in DNA: A Density Functional Theory (DFT) Study. *Int. J. Mol. Sci.* **2021**, *22*, 1736; https://doi.org/10.3390/ijms22041736 (Ref. **216** in the References list to the given article)

259. Maria Chiara di Gregorio, Jacopo Cautela and Luciano Galantini. Physiology and Physical Chemistry of Bile Acids. *Int. J. Mol. Sci.* **2021**, *22*, 1780; https://doi.org/10.3390/ijms22041780 (Ref. **291** in the References list to the given article)

260. Theodora Christoforidou, Dimitra Giasafaki, Eleftherios G. Andriotis, Nikolaos Bouropoulos, Nikoleta F. Theodoroula, Ioannis S. Vizirianakis, Theodore Steriotis, Georgia Charalambopoulou and Dimitrios G. Fatouros. Oral Drug Delivery Systems Based on Ordered Mesoporous Silica Nanoparticles for Modulating the Release of Aprepitant. Int. J. Mol. Sci. 2021, 22(4), 1896; https://doi.org/10.3390/ijms22041896 (Ref. **97** in the References list to the given article)

261. Claire Egloff-Juras, Ilya Yakavets, Victoria Scherrer, Aurélie Francois, Lina Bezdetnaya, Henri-Pierre Lassalle and Gilles Dolivet. Validation of a Three-Dimensional Head and Neck Spheroid Model to Evaluate Cameras for NIR Fluorescence-Guided Cancer Surgery. *Int. J. Mol. Sci.* **2021**, *22*, 1966; https://doi.org/10.3390/ijms22041966 (Ref. **180** in the References list to the given article)

262. Fuyuhiko Tamanoi, Shanmugavel Chinnathambi, Mathilde Laird, Aoi Komatsu, Albane Birault, Takushi Takata, Tan Le-Hoang Doan, Ngoc Xuan Dat Mai, Arthur Raitano, Kendall Morrison, Minoru Suzuki and Kotaro Matsumoto. Construction of Boronophenylalanine-Loaded Biodegradable Periodic Mesoporous

Organosilica Nanoparticles for BNCT Cancer Therapy. *Int. J. Mol. Sci.* **2021**, *22*, 2251; https://doi.org/10.3390/ijms22052251 (Ref. **98** in the References list to the given article)

263. Eugene Arthur-Baidoo, Karina Falkiewicz, Lidia Chomicz-Mańka, Anna Czaja, Sebastian Demkowicz, Karol Biernacki, Witold Kozak, Janusz Rak and Stephan Denifl. Electron-Induced Decomposition of Uracil-5-yl O-(N,N-dimethylsulfamate): Role of Methylation in Molecular Stability. *Int. J. Mol. Sci.* **2021**, *22*, 2344; https://doi.org/10.3390/ijms22052344 (Ref. **250** in the References list to the given article)

264. Sergio Giuffrida, Antonio Cupane and Grazia Cottone. "Water Association" Band in Saccharide Amorphous Matrices: Role of Residual Water on Bioprotection. *Int. J. Mol. Sci.* **2021**, *22*, 2496; https://doi.org/10.3390/ijms22052496 (Ref. **181** in the References list to the given article)

265. Sung-Won Shin, Kyungmi Yang, Miso Lee, Jiyoung Moon, Arang Son, Yeeun Kim, Suha Choi, Do-hyung Kim, Changhoon Choi, Nohyun Lee and Hee Chul Park. Manganese Ferrite Nanoparticles Enhance the Sensitivity of Hepa1-6 Hepatocellular Carcinoma to Radiation by Remodeling Tumor Microenvironments. *Int. J. Mol. Sci.* **2021**, *22*, 2637; https://doi.org/10.3390/ijms22052637 (Ref. **292** in the References list to the given article)

266. Vladimir Shafirovich and Nicholas E. Geacintov. Excision of Oxidatively Generated Guanine Lesions by Competitive DNA Repair Pathways. *Int. J. Mol. Sci.* **2021**, *22*, 2698; https://doi.org/10.3390/ijms22052698 (Ref. **293** in the References list to the given article)

267. Andrea Correa, Antonio De Nicola, Giuseppe Scherillo, Valerio Loianno, Domenico Mallamace, Francesco Mallamace, Hiroshi Ito, Pellegrino Musto and Giuseppe Mensitieri. A Molecular Interpretation of the Dynamics of Diffusive Mass Transport of Water within a Glassy Polyetherimide. *Int. J. Mol. Sci.* **2021**, *22*, 2908; https://doi.org/10.3390/ijms22062908 (Ref. **23** in the References list to the given article)

268. Monika Halat, Magdalena Klimek-Chodacka, Jagoda Orleanska, Malgorzata Baranska and Rafal Baranski. Electronic Circular Dichroism of the Cas9 Protein and gRNA:Cas9 Ribonucleoprotein Complex. *Int. J. Mol. Sci.* **2021**, *22*, 2937; https://doi.org/10.3390/ijms22062937 (Ref. **294** in the References list to the given article)

269. Andrey A. Shtyrov, Dmitrii M. Nikolaev, Vladimir N. Mironov, Andrey V. Vasin, Maxim S. Panov, Yuri S. Tveryanovich and Mikhail N. Ryazantsev. Simple Models to Study Spectral Properties of Microbial and Animal Rhodopsins: Evaluation of the Electrostatic Effect of Charged and Polar Residues on the First Absorption Band Maxima. *Int. J. Mol. Sci.* **2021**, *22*, 3029; https://doi.org/10.3390/ijms22063029 (Ref. **217** in the References list to the given article)

270. Eugene Arthur-Baidoo, Joao Ameixa, Milan Ončák and Stephan Denifl. Ring-Selective Fragmentation in the Tirapazamine Molecule upon Low-Energy Electron Attachment. *Int. J. Mol. Sci.* **2021**, *22*, 3159; https://doi.org/10.3390/ijms22063159 (Ref. **251** in the References list to the given article)

271. Celia Nieto, Milena A. Vega and Eva M. Martín del Valle. Tailored-Made Polydopamine Nanoparticles to Induce Ferroptosis in Breast Cancer Cells in Combination with Chemotherapy. *Int. J. Mol. Sci.* **2021**, *22*, 3161; https://doi.org/10.3390/ijms22063161 (Ref. **182** in the References list to the given article)

272. Lucija Hok and Robert Vianello. Direct Metal-Free Transformation of Alkynes to Nitriles: Computational Evidence for the Precise Reaction Mechanism. *Int. J. Mol. Sci.* **2021**, *22*, 3193; https://doi.org/10.3390/ijms22063193 (Ref. **218** in the References list to the given article)

273. Jiří Czernek and Jiří Brus. A Volumetric Analysis of the 1H NMR Chemical Shielding in Supramolecular Systems. *Int. J. Mol. Sci.* **2021**, *22*, 3333; https://doi.org/10.3390/ijms22073333 (Ref. **183** in the References list to the given article)

274. Magdalena Włodarska and Beata Mossety-Leszczak. DFT Studies of Selected Epoxies with Mesogenic Units–Impact of Molecular Structure on Electro-Optical Response. *Int. J. Mol. Sci.* **2021**, *22*, 3424; https://doi.org/10.3390/ijms22073424 (Ref. **219** in the References list to the given article)

275. Anton Muravev, Ayrat Yakupov, Tatiana Gerasimova, Ramil Nugmanov, Ekaterina Trushina, Olga Babaeva, Guliya Nizameeva, Viktor Syakaev, Sergey Katsyuba, Sofiya Selektor, Svetlana Solovieva and Igor Antipin. Switching Ion Binding Selectivity of Thiacalix[4]arene Monocrowns at Liquid–Liquid and 2D-Confined Interfaces. *Int. J. Mol. Sci.* **2021**, *22*, 3535; https://doi.org/10.3390/ijms22073535 (Ref. **184** in the References list to the given article)

276. Victor Nikonenko and Natalia Pismenskaya. Ion and Molecule Transport in Membrane Systems 2.0. *Int. J. Mol. Sci.* **2021**, *22*, 3533; https://doi.org/10.3390/ijms22073533 (Ref. **99** in the References list to the given article)

277. Victor Nikonenko and Natalia Pismenskaya. Ion and Molecule Transport in Membrane Systems. *Int. J. Mol. Sci*. **2021**, *22*, 3556; https://doi.org/10.3390/ijms22073556 (Ref. **100** in the References list to the given article)

278. Md Afjalus Siraj, Md. Sajjadur Rahman, Ghee T. Tan and Veronique Seidel. Molecular Docking and Molecular Dynamics Simulation Studies of Triterpenes from Vernonia patula with the Cannabinoid Type 1 Receptor. *Int. J. Mol. Sci*. **2021**, *22*, 3595; https://doi.org/10.3390/ijms22073595 (Ref. **185** in the References list to the given article)

279. Anna Sowińska, Magdalena Maciejewska and Anna Grajewska. Bis(trifluoromethylsulfonyl)imide Ionic Liquids Applied for Fine-Tuning the Cure Characteristics and Performance of Natural Rubber Composites. *Int. J. Mol. Sci*. **2021**, *22*, 3678; https://doi.org/10.3390/ijms22073678 (Ref. **58** in the References list to the given article)

280. Oliver Schmutzler, Sebastian Graf, Nils Behm, Wael Y. Mansour, Florian Blumendorf, Theresa Staufer, Christian Körnig, Dina Salah, Yanan Kang, Jan N. Peters, Yang Liu, Neus Feliu, Wolfgang J. Parak, Anja Burkhardt, Elisabetta Gargioni, Sabrina Gennis, Sharah Chandralingam, Finn Höeg, Wolfgang Maison, Kai Rothkamm, Florian Schulz and Florian Grüner. X-ray Fluorescence Uptake Measurement of Functionalized Gold Nanoparticles in Tumor Cell Microsamples. *Int. J. Mol. Sci*. **2021**, *22*, 3691; https://doi.org/10.3390/ijms22073691 (Ref. **295** in the References list to the given article)

281. Ayrat R. Khamatgalimov and Valeri I. Kovalenko. Substructural Approach for Assessing the Stability of Higher Fullerenes. *Int. J. Mol. Sci*. **2021**, *22*, 3760; https://doi.org/10.3390/ijms22073760 (Ref. **220** in the References list to the given article)

282. Elisa Poyatos-Racionero, Gemma Guarí-Borràs, María Ruiz-Rico, Ángela Morellá-Aucejo, Elena Aznar, José Manuel Barat, Ramón Martínez-Máñez, María Dolores Marcos and Andrea Bernardos. Towards the Enhancement of Essential Oil Components' Antimicrobial Activity Using New Zein Protein-Gated Mesoporous Silica Microdevices. *Int. J. Mol. Sci*. **2021**, *22*, 3795; https://doi.org/10.3390/ijms22073795 (Ref. **296** in the References list to the given article)

283. Ji Young Lee, Changhoon Lee, Eiji Osawa, Jong Woan Choi, Jung Chul Sur and Kee Hag Lee. Snapshots of the Fragmentation for C70@Single-Walled Carbon Nanotube: Tight-Binding Molecular Dynamics Simulations. *Int. J. Mol. Sci*. **2021**, *22*, 3929; https://doi.org/10.3390/ijms22083929 (Ref. **297** in the References list to the given article)

284. Jianhua Zhang, Esther Wehrle, Marina Rubert and Ralph Müller. 3D Bioprinting of Human Tissues: Biofabrication, Bioinks, and Bioreactors. *Int. J. Mol. Sci*. **2021**, *22*, 3971; https://doi.org/10.3390/ijms22083971 (Ref. **298** in the References list to the given article)

285. Silvia Franco, Elena Buratti, Valentina Nigro, Emanuela Zaccarelli, Barbara Ruzicka and Roberta Angelini. Glass and Jamming Rheology in Soft Particles Made of PNIPAM and Polyacrylic Acid. *Int. J. Mol. Sci*. **2021**, *22*, 4032; https://doi.org/10.3390/ijms22084032 (Ref. **24** in the References list to the given article)

286. Lajos Nagy, Bence Vadkerti, Csilla Lakatos, Péter Pál Fehér, Miklós Zsuga and Sándor Kéki. Kinetically Equivalent Functionality and Reactivity of Commonly Used Biocompatible Polyurethane Crosslinking Agents. *Int. J. Mol. Sci*. **2021**, *22*, 4059; https://doi.org/10.3390/ijms22084059 (Ref. **47** in the References list to the given article)

287. Michael Filatov, Seunghoon Lee, Hiroya Nakata and Cheol-Ho Choi. Signatures of Conical Intersection Dynamics in the Time-Resolved Photoelectron Spectrum of Furan: Theoretical Modeling with an Ensemble Density Functional Theory Method. *Int. J. Mol. Sci*. **2021**, *22*, 4276; https://doi.org/10.3390/ijms22084276 (Ref. **221** in the References list to the given article)

288. Anna Helena Mazurek, Łukasz Szeleszczuk and Dariusz Maciej Pisklak. A Review on Combination of Ab Initio Molecular Dynamics and NMR Parameters Calculations. *Int. J. Mol. Sci*. **2021**, *22*, 4378; https://doi.org/10.3390/ijms22094378 (Ref. **222** in the References list to the given article)

289. Marie Hullo, Romain Grall, Yann Perrot, Cécile Mathé, Véronique Ménard, Xiaomin Yang, Sandrine Lacombe, Erika Porcel, Carmen Villagrasa, Sylvie Chevillard and Emmanuelle Bourneuf. Radiation Enhancer Effect of Platinum Nanoparticles in Breast Cancer Cell Lines: In Vitro and In Silico Analyses. *Int. J. Mol. Sci*. **2021**, *22*, 4436; https://doi.org/10.3390/ijms22094436 (Ref. **299** in the References list to the given article)

290. Ana I. Lozano, Filipe Costa, Xueguang Ren, Alexander Dorn, Lidia Álvarez, Francisco Blanco, Paulo Limão-Vieira and Gustavo García. Double and Triple Differential Cross Sections for Single Ionization of Benzene by

Electron Impact. *Int. J. Mol. Sci*. **2021**, *22*, 4601; https://doi.org/10.3390/ijms22094601 (Ref. **232** in the References list to the given article)

291. Paulina Laszuk and Aneta D. Petelska. Interactions between Phosphatidylcholine and Kaempferol or Myristicin: Langmuir Monolayers and Microelectrophoretic Studies. *Int. J. Mol. Sci*. **2021**, *22*, 4729; https://doi.org/10.3390/ijms22094729 (Ref. **101** in the References list to the given article)

292. Tomasz Pędzinski, Katarzyna Grzyb, Konrad Skotnicki, Piotr Filipiak, Krzysztof Bobrowski, Chryssostomos Chatgilialoglu and Bronislaw Marciniak. Radiation- and Photo-Induced Oxidation Pathways of Methionine in Model Peptide Backbone under Anoxic Conditions. *Int. J. Mol. Sci*. **2021**, *22*, 4773; https://doi.org/10.3390/ijms22094773 (Ref. **233** in the References list to the given article)

293. Eduardo Gomez, Ichiro Hisaki and Abderrazzak Douhal. Synthesis and Photobehavior of a New Dehydrobenzoannulene-Based HOF with Fluorine Atoms: From Solution to Single Crystals Observation. *Int. J. Mol. Sci*. **2021**, *22*, 4803; https://doi.org/10.3390/ijms22094803 (Ref. **234** in the References list to the given article)

294. Edyta Pindelska, Anna Marczewska-Rak, Jolanta Jaśkowska and Izabela D. Madura. Solvates of New Arylpiperazine Salicylamide Derivative – a Multi-Technique Approach to the Description of 5 HTR Ligand Structure and Interactions. *Int. J. Mol. Sci*. **2021**, *22*, 4992; https://doi.org/10.3390/ijms22094992 (Ref. **186** in the References list to the given article)

295. Xiaoyong Cao and Pu Tian. "Dividing and Conquering" and "Caching" in Molecular Modeling. *Int. J. Mol. Sci*. **2021**, *22*, 5053; https://doi.org/10.3390/ijms22095053 (Ref. **187** in the References list to the given article)

296. Dipankar Roy and Andriy Kovalenko. Biomolecular Simulations with the Three-Dimensional Reference Interaction Site Model with the Kovalenko-Hirata Closure Molecular Solvation Theory. *Int. J. Mol. Sci*. **2021**, *22*, 5061; https://doi.org/10.3390/ijms22105061 (Ref. **188** in the References list to the given article)

297. Daniel Lach, Uladzislau Zhdan, Adam Smolinski and Jaroslaw Polanski. Functional and Material Properties in Nanocatalyst Design: A Data Handling and Sharing Problem. *Int. J. Mol. Sci*. **2021**, *22*, 5176; https://doi.org/10.3390/ijms22105176 (Ref. **223** in the References list to the given article)

298. Andrzej Bak. Two Decades of 4D-QSAR: A Dying Art or Staging a Comeback? *Int. J. Mol. Sci*. **2021**, *22*, 5212; https://doi.org/10.3390/ijms22105212 (Ref. **189** in the References list to the given article)

299. Jarosław J. Panek, Joanna Zasada, Bartłomiej M. Szyja, Beata Kizior and Aneta Jezierska. Sensitivity of Intra- and Intermolecular Interactions of Benzo[h]quinoline from Car–Parrinello Molecular Dynamics and Electronic Structure Inspection. *Int. J. Mol. Sci*. **2021**, *22*, 5220; https://doi.org/10.3390/ijms22105220 (Ref. **25** in the References list to the given article)

300. Sean R. Tachibana, Longteng Tang, Liangdong Zhu, Yuka Takeda, Keiji Fushimi, Yoshibumi Ueda, Takahiro Nakajima, Yuto Kuwasaki, Moritoshi Sato, Rei Narikawa and Chong Fang. An Engineered Biliverdin-Compatible Cyanobacteriochrome Enables a Unique Ultrafast Reversible Photoswitching Pathway. *Int. J. Mol. Sci*. **2021**, 22, 5252; https://doi.org/10.3390/ijms22105252 (Ref. **300** in the References list to the given article)

© 2020 by the authors. Submitted for possible open access publication under the terms and conditions of the Creative Commons Attribution (CC BY) license (http://creativecommons.org/licenses/by/4.0/).
